# Structural dynamics influences the antibacterial activity of a cell-penetrating peptide (KFF)_3_K

**DOI:** 10.1038/s41598-023-38745-y

**Published:** 2023-09-08

**Authors:** Julia Macyszyn, Piotr Chyży, Michał Burmistrz, Małgorzata Lobka, Joanna Miszkiewicz, Monika Wojciechowska, Joanna Trylska

**Affiliations:** 1 Centre of New Technologies, University of Warsaw, Warsaw, Poland; 2 Division of Biophysics, Institute of Experimental Physics, Faculty of Physics, University of Warsaw, Warsaw, Poland; 3 College of Inter-Faculty Individual Studies in Mathematics and Natural Sciences, University of Warsaw, Warsaw, Poland

**Keywords:** Antimicrobials, Biochemistry, Peptides, Computational biophysics, Computational chemistry, Peptides, Peptides

## Abstract

Given the widespread demand for novel antibacterial agents, we modified a cell-penetrating peptide (KFF)_3_K to transform it into an antibacterial peptide. Namely, we inserted a hydrocarbon staple into the (KFF)_3_K sequence to induce and stabilize its membrane-active secondary structure. The staples were introduced at two positions, (KFF)_3_K[5–9] and (KFF)_3_K[2–6], to retain the initial amphipathic character of the unstapled peptide. The stapled analogues are protease resistant contrary to (KFF)_3_K; 90% of the stapled (KFF)_3_K[5–9] peptide remained undigested after incubation in chymotrypsin solution. The stapled peptides showed antibacterial activity (with minimal inhibitory concentrations in the range of 2–16 µM) against various Gram-positive and Gram-negative strains, contrary to unmodified (KFF)_3_K, which had no antibacterial effect against any strain at concentrations up to 32 µM. Also, both stapled peptides adopted an α-helical structure in the buffer and micellar environment, contrary to a mostly undefined structure of the unstapled (KFF)_3_K in the buffer. We found that the antibacterial activity of (KFF)_3_K analogues is related to their disruptive effect on cell membranes and we showed that by stapling this cell-penetrating peptide, we can induce its antibacterial character.

## Introduction

Membrane-active peptides can destabilize or penetrate across the cell membrane^1^. Two classes of membrane-active peptides are typically distinguished: Antimicrobial Peptides (AMP) and Cell Penetrating Peptides (CPP). The major difference is that AMP inhibit the cellular functions of bacteria, viruses, fungi or cancer cells mainly by disrupting membrane integrity^2^. Contrary, CPP can cross plasma membranes while maintaining their integrity, so they are not antimicrobial compounds themselves^3–5^.

In general, CPP are short peptides (< 30 amino acids) that can intracellularly deliver bioactive molecules such as drugs, oligonucleotides, and proteins^6–9^. They include cationic, amphipathic and hydrophobic peptides with the majority adopting a random coil conformation in solution^10–12^. The efficiency of CPP uptake across the membrane and their transport potential depend on CPP physicochemical properties, the nature of the cargo molecule, the ability of CPP to adopt membrane-active secondary structure, and the composition of the membrane^13–17^. In general, the amphipathic and cationic peptides are better membrane permeabilizers than solely hydrophobic ones^12^. For example, a cationic, arginine-rich peptide, an analog of the HIV-1 trans-activator of transcription protein, showed the highest penetration efficiency into the *Escherichia coli* (*E. coli*) DH5α strain^12^.

We focus on the (KFF)_3_K peptide, first synthesized by Vaara and Porro in 1996 based on the backbone of polymyxin B^18^. (KFF)_3_K is an amphipathic and cationic CPP that exhibits poor antibacterial activity; its minimal inhibitory concentration (MIC) against *E. coli* K12 MG1655 is 32 µM^19^. In addition, the MIC against the Gram-positive *Staphylococcus aureus* (*S. aureus*) ATCC 29213 is above 60 µM^20^. Therefore, (KFF)_3_K does not have the inherent antimicrobial activity and cannot be considered an AMP. However, (KFF)_3_K was found to facilitate the uptake of other antibiotics by destabilizing the outer membrane without disrupting the cytoplasmic membrane^18^. Because of this, (KFF)_3_K has been widely used as a transporter of peptide nucleic acids (PNA) into both Gram-negative and Gram-positive bacteria^21–25^. For example, (KFF)_3_K was used as a carrier of a PNA oligomer targeting the transcript encoding the essential acyl carrier protein. The (KFF)_3_K-mediated PNA transport to the cytoplasm allowed PNA binding to this transcript and induced an antibacterial effect against the *E. coli* K12 strain at PNA concentrations on the order of 1 µM^24^. Unfortunately, the PNA uptake efficiency depends on the peptide part of the conjugate and (KFF)_3_K is unstable and degraded by the peptidases^26^.

Typically, CPP cross the membranes using two main mechanisms: endocytosis or direct penetration, which depend on the cell type and the nature of the peptide^27^. For cationic CPP with arginine-rich regions, endocytosis is the preferable mode of intracellular transport^28^. In contrast, amphipathic or hydrophobic CPP prefer direct penetration by either pore-forming or a carpet model^3,29^. In particular, the amphipathic CPP interact with the membrane, destabilizing it in the same way as AMP^30^. On these grounds, CPP sometimes behave similarly to AMP, making them difficult to distinguish^31^.

Furthermore, a single-point mutation of a peptide sequence can change its solely cell-penetrating ability into antimicrobial activity, or vice versa. It was shown that the incorporation of hydrophobic or cationic residues changed the character of a CPP into a more antibacterial one. For example, replacing Glu with Lys in the Pep-1 CPP (KETWWETWWTEWSQPKKKRKV with high MIC of 32–64 µM) induced its antimicrobial activity and lowered MIC to 1–2 µM for both Gram-positive and Gram-negative strains^32,33^.

Essentially, the mechanism of action of most AMP involves their interaction with the bacterial membrane, damage to the lipid bilayer by pore formation, and the resulting loss of membrane continuity^34^. The cationic region facilitates the initial membrane contact, while the hydrophobic region impacts transport across the lipid bilayer. Such amphipathic peptides are often structurally disordered in aqueous solution and only adopt an active secondary structure (a helix, a β-turn or a β-sheet) in the lipid environment^19,35^. An annexin protein N-terminus isoform (AAH3, MASIWVGHRG) is an example of a CPP with some β-sheet structure in the buffer solution that provides enhanced cell membrane permeability^36^. A helical structure of the amphipathic Pep-1 peptide proved to be essential for its bactericidal activity^32,33^. Therefore, to induce or enhance the antibacterial activity of a membrane-active peptide it seems necessary to induce formation or stabilize its active secondary structure that promotes cell membrane destabilization. Stabilization of the active structure reduces the entropic cost that the peptide would have to pay to interact efficiently with the membrane, making it a better antibacterial agent.

Simultaneously, reducing peptide susceptibility to proteolytic degradation is also crucial^27^. Reports showed that introducing D-amino acids into the KKVVFKVKFKK peptide at the N- or C-terminus increased its half-life by 2- or threefold, without significantly disturbing its tendency to form the secondary structure^37^. However, apart from improved proteolytic stability, the introduction of terminal D-amino acids did not significantly increase the antibacterial activity of the peptide. On the other hand, the hydrocarbon stapling technique introduced by Schafmeister et al*.* in 2000, not only aimed at increasing the proteolytic stability of the peptide but also at stabilizing its active secondary structure^38,39^.

The idea of peptide stapling is based on replacing two residues with α,α-disubstituted amino acids bearing olefinic chains at positions *i*, *i* + *4* or *i, i* + *7* (where "*i*" represents the first staple position). The staple - a covalent hydrocarbon bridge - is formed by a ring-closing metathesis reaction^40^. In some cases, longer peptides could also be double-stapled^38,41^. Hydrocarbon-stapled peptides are widely used in biological applications, such as anticancer, antiviral or antimicrobial^42^. It was shown that the insertion of a hydrocarbon staple into antimicrobial peptides enhances their activity by stabilizing their secondary structures^39,43,44^. Luong et al. demonstrated that hydrocarbon stapling of Polybia-MP1 (IDWKKLLDAAKQIL-*NH*_*2*_) resulted in a more active against Gram-positive bacteria and metabolically stable analog than the natural one^45^. Moreover, Mourtada et al. designed a selective double-stapled analog of the 23-amino acid peptide magainin II, which showed up to four-fold improved potency against Gram-negative strains compared to the single-stapled analog^46^.

We have also previously shown that hydrocarbon stapling of a membrane-active secondary structure of a natural AMP, anoplin, improves its proteolytic stability, membrane disruption and, importantly, antibacterial activity^47^. Furthermore, we have also shown that amphipathic peptides, including (KFF)_3_K, change their secondary structure in the presence of various membrane mimics^19^. Therefore, intending to transform the CPP into an AMP without compromising its low hemolytic activity, we modified the (KFF)_3_K peptide. We hydrocarbon stapled this peptide to stabilize its secondary structure supposedly in a form, which has a natural ability to cross the bacterial cell membrane. We tested if introducing the staple into the (KFF)_3_K sequence enhances its proteolytic stability. We applied experimental and simulation methods to characterize the structure of this peptide in the buffer solution and micellar environment. We further determined whether these modifications increase the membrane-destabilizing effect and induce antibacterial properties of (KFF)_3_K, and at the same time do not increase the hemolytic activity on sheep erythrocytes.

## Materials and methods

### Peptide synthesis and purification

All peptides were synthesized manually with the Fmoc (9-fluorenylmetoxycarbonyl) chemistry solid-phase peptide synthesis on the Rink-amide resin (Tenta Gel S RAM, amine groups loading of 0.24 mmol/g; this resin has a linker which yields a C-terminal amine upon trifluoroacetic acid cleavage of the peptide)^48^. The coupling of unmodified and modified (KFF)_3_K analogs was carried out with threefold molar excess of Fmoc-protected amino acids using a threefold molar excess of the activator reagents: *O*-(7-Aza-1*H*-benzotriazole-1-yl)-*N*,*N*,*N*′,*N*′-tetramethyluronium hexafluorophosphate (HATU) and 1-hydroxy-7-azabenzotriazole (HOAt), a sixfold molar excess of collidine and *N*,*N*-Dimethylpyridin-4-amine as a catalyst, whole dissolved in the dimethylformamide/*N*-methylpyrrolidone (DMF/ NMP) (1:1, v:v) as a solution and mixed for 1.5 h. The reaction progress of each step was confirmed by the negative result of the Kaiser test^49^. The positive test result confirmed the necessity of repeating the reaction with half-reduced activating reagents. Whenever the coupling of (S)-2-(4′-pentenyl)-alanine (PEN) in the peptide sequence was not successful, the activation method was repeated. The Fmoc deprotection was accomplished using 20% piperidine in DMF for 2 cycles through 10 min. Ring-closing metathesis (RCM) was performed using a 0.25-fold molar excess of the 1st Generation of Grubbs Catalyst dissolved in degassed 1,2-dichloroethane. The solution and resin were stirred at room temperature for 2 h under the nitrogen atmosphere^39^. The metathesis reaction was repeated three times with a fresh portion of Grubbs catalyst to complete the reaction. Finally, the Fmoc protecting group was removed from the last N-terminal amino acid after the RCM reaction. The peptide was deprotected and cleaved from the resin by treatment with a trifluoroacetic acid/triisopropylsilane/water (95:2.5:2.5; v/v/v) mixture for 2 h.

The synthesized peptides were analyzed and purified by analytical and semi-preparative reverse-phase high-performance liquid chromatography (RP-HPLC) (Knauer C18 columns, 5 µM particles, 4.6 × 250 mm and 8 × 250 mm, respectively). Peptides were purified using buffer A (0,1% trifluoroacetic acid in water) and buffer B (0,1% trifluoroacetic acid in acetonitrile) at a flow rate of 1.5 ml/min and wavelength 220 nm. The mobile phase gradient used was from 15 to 70% buffer B for 30 min. The purity and identity of the peptides were checked by RP-HPLC and mass spectrometry using the Q-TOF Premier mass spectrometer. To exchange the trifluoroacetic anion for hydrochloride before the spectral measurements, peptides were dissolved in a 0.1 M HCl solution, frozen, and lyophilized.

### Circular dichroism spectroscopy

Circular dichroism (CD) spectra of peptides at concentrations of 120 µM were recorded at room temperature in 10 mM phosphate buffer (pH 7) and the presence of 5 mM sodium dodecyl sulfate (SDS) or 2 mM dodecylphosphocholine (DPC) micelles as previously described^19^. The Biokine MOS-450/AF-CD spectrometer equipped with the Xe lamp with a 0.1 cm CD path length cuvette was used. The acquisition duration time was 2 s with a resolution of 1 nm over the wavelength range 190–260 nm. Contributions from micelles were eliminated by subtracting their spectra from the corresponding peptide spectra with micelles. The CD spectra are the averages of three scans, were smoothed with the Savitzky–Golay method and shown using GraphPad. Every CD experiment was conducted twice. The high-tension values, showing excessive light scattering or absorption of light, were below 600 V. The percentage of a helix (regular and disordered), β-sheet (regular and disordered), turns or unordered structure in CD spectra was estimated based on the DichroWeb software^50^. The spectra were analyzed with the CDSSTR program and DataSet4 data^51,52^. The percentages are averages with SEM from two independent CD experiments and are summarized in Table 1.

**Table 1:** The percentage of a helix (regular and disordered), β-sheet (regular and disordered), turns and unordered (or other) structures in the CD spectra calculated with DichroWeb. For (KFF)_3_K the data in the SDS solution were taken from Wojciechowska et al.^19^.

	Helix (%)	β-sheet (%)	Turns (%)	Unordered (%)
Phosphate buffer				
(KFF)_3_K	34.2 ± 6.2	33.2 ± 3.9	12.6 ± 3.5	20.1 ± 1.1
(KFF)_3_K[2–6]	37.6 ± 4.9	27.5 ± 0.2	17.5 ± 7.4	17.6 ± 2.7
(KFF)_3_K[5–9]	45.8 ± 1.8	25.7 ± 0.4	9.6 ± 0.5	19.1 ± 0.9
SDS [5 mM]				
(KFF)_3_K	44.5 ± 0.5	28.0 ± 0.0	8.5 ± 0.5	17.0 ± 4.0
(KFF)_3_K[2–6]	56.5 ± 7.0	19.0 ± 3.2	8.5 ± 0.6	16.1 ± 3.2
(KFF)_3_K[5–9]	55.5 ± 5.5	19.0 ± 3.0	6.0 ± 2.0	19.5 ± 0.5
DPC [2 mM]				
(KFF)_3_K	24.8 ± 0.0	28.7 ± 0.0	7.9 ± 0.0	38.6 ± 0.0
(KFF)_3_K[2–6]	57.5 ± 2.5	16.5 ± 2.5	8.0 ± 1.0	18.0 ± 1.0
(KFF)_3_K[5–9]	61.8 ± 1.8	16.1 ± 1.1	6.1 ± 0.1	16.1 ± 3.0

### Molecular dynamics simulations

The initial structures of the (KFF)_3_K peptides and (KFF)_3_K/SDS complexes were generated by LEaP from Amber20^53^. For consistency with the synthesized peptides, the positively charged N-terminus (NH_3_^+^) and amidated C-terminus (CONH_2_) were applied. All lysines were protonated. In simulations starting from the helical structure of the peptide, the φ (C_i-1_–N_i_–CA_i_–C_i_) and ψ (N_i_–CA_i_–C_i_–N_i+1_) angles were set to −57° and −47°, respectively. For stapled peptides, the structure of 2-(4′-pentenyl)-alanine (PEN) was taken from^54^, but the CA configuration was modified to be compatible with the synthesized peptides. To reflect the ring-closing metathesis, the CZ group hydrogens in the PEN residues were removed, and the CZ atom was replaced by hydrogen, corresponding to the HE atom. To neutralize the charge of PEN after ring-closing, the partial charge of the removed CZ group was added to the CE atom. Finally, to create the staple in the *cis* configuration, the HE atom was removed, allowing for linking the CE atoms via a double bond^44,54^.

The micelles were built in the CHARMM-GUI^55,56^ from 60 SDS detergents to reflect the experimentally determined aggregation number^57–59^ and previous molecular dynamics simulations of the SDS molecules^60^. Solutes were embedded in a rectangular simulation box with periodic boundary conditions and were solvated by adding TIP3P water molecules providing a 15 Å-thick water shell. In the simulations with the SDS micelle, the peptides were simulated from two starting positions, either perpendicular or parallel to the SDS surface. The closest atoms were about 5–6 Å from the micelle surface (either the peptide N-terminus or Lys4/Lys7 depending on the starting position). The (KFF)_3_K solution with the phosphate buffer contains 1 H_2_PO_4_^−^, 1 HPO_4_^2−^, and 3 Na^+^ molecules, together with 5 Cl^-^ ions to neutralize the peptide^53^. For systems with a micelle, 69 Na^+^, 3 H_2_PO_4_^−^, 3 HPO_4_^2−^, and 5 Cl^−^ molecules were added. The ff14SB, TIP3P, and IONSJC_TIP3P Amber force field parameters were applied for standard residues, water molecules, and Na^+^ and Cl^−^ ions^61–63^. The RESP charges for the PEN residue, the SDS detergent, and the H_2_PO_4_^−^ and HPO_4_^2−^ molecules were taken from the literature^54,60,64,65^.

Calculations were carried out with the PMEMD from Amber 20^53^. The particle mesh Ewald method, with an 8 Å cutoff distance, was implemented to calculate long-range electrostatic interactions^53^. Hydrogen mass repartitioning was used to increase the mass of hydrogen and increase the simulation timestep to 4 fs^66^. The SHAKE algorithm was applied to keep rigid the water molecules and bonds with hydrogen atoms^53^. Two energy minimizations were performed (each with 5000 steps of the steepest descent algorithm followed by 500 steps of the conjugate gradient algorithm): first, with the positional restraints of 50 kcal/mol/Å^2^ set on heavy atoms of the solute to relax the created staple, and, second, with restraints decreased to 10 kcal/mol/Å^2^. Next, the systems were gradually heated from 0.15 K to 310.15 K with the Langevin thermostat and the same positional restraints as in the previous step^53^. During the following equilibration, in the NVT ensemble, the superimposed positional restraints were gradually decreased. Before the production, the 20 ns equilibration in the NPT ensemble, with a Monte Carlo barostat and without any restraints, was performed^53^. The production phases, in the NPT ensemble, lasted 500 ns each and were run in three replicates. The DSSP algorithm, implemented in CPPTRAJ, was used for secondary structure analysis^67,68^. Based on the root-mean-square deviation (RMSD) values, the K-means clustering algorithm was applied to find the 10 most probable structures of the peptide in the entire trajectory. Matplotlib and Visual Molecular Dynamics were used to visualize the simulation results^68–70^.

### Stability assay

The proteolytic stability of peptides was analyzed against α-chymotrypsin from bovine pancreas (3.4.21.1, ≥ 40 units/mg protein). Peptide solutions (340 µL) in ammonium bicarbonate buffer (0.1 M NH_4_HCO_3_, pH 8.0) were incubated with the appropriate amount of chymotrypsin at 37 ºC with rapid shaking (600 rpm). Chymotrypsin and peptides were mixed in the 1:1333 ratio^47,71^. Digestion mixture (60 µL) was taken at 4, 8, 12, 16, and 20 min and treated with 3 µL of 50% acetonitrile containing 1% TFA to stop the reaction and immediately store in ice. Peptide degradation was analyzed with RP-HPLC using the analytical method from 15 to 75% of acetonitrile in 30 min. Before the injection, the sample was diluted by adding 57 µL of ammonium bicarbonate buffer. 100 µL of the solution taken in each period was injected. The stabilities were quantified as the percentage of the peptide peak of area degradation. The total area of each peak was determined for experiments without chymotrypsin. Each experiment was performed twice on different days.

### Propidium iodide uptake assay

The propensity of peptides to disrupt and destabilize the membrane integrity was measured by the propidium iodide (PI) uptake assay. The bacterial strains of *E. coli* K12 MG1655 and *S. aureus* ATCC 25923 were grown in Mueller Hinton Broth (MHB, Difco) until the optical density (OD) at 600 nm reached 0.27. Next, appropriate concentrations of peptides (128, 64, 32, 16, 8, 4, 2 µM) diluted in the MHB media and 50 μL of the bacterial suspension containing PI (final concentration of 10 μM) were added to the wells of a black-walled microplate. Untreated bacteria in the MHB solution were used as negative controls. The emitted fluorescence was measured at the excitation wavelength of 580 nm and emission wavelength of 610 nm on a Microplate Reader Biotek (Winooski, VT, USA). The microplate was incubated for 2 h at 37 °C. The scan was performed every 1 min. The normalized fluorescence intensity induced by the peptides was calculated using the following equation:

$$Normalized\,fluorescence\,intensity=\frac{\left(F-{F}_{0}\right)}{\left({F}_{100}-{F}_{0}\right)}$$

where F_0_ is the fluorescence intensity of the control bacterial cells in untreated PI solution, and F and F_100_ are the fluorescence intensities of the peptides at the tested PI concentration and the peptides at the concentration of 64 µM, respectively^71^. The experiment was performed in triplicates.

### Antibacterial activity determination

The minimal inhibitory concentration (MIC) values were determined as follows. Bacteria were first cultured overnight in 2 mL of Lysogeny Broth (LB, VWR Chemicals) at 37 °C with shaking. Second, 20 µL of overnight culture was transferred into 2 mL of MHB medium and further cultured at 37 °C with shaking until the culture reached OD_600_ of 0.3. Third, the culture was diluted 1:100 in a fresh MHB medium and aliquots of 50 µL were mixed with 50 µL of the previously prepared dilutions of the tested compound in MHB on a transparent flat-bottom 96-well plate (Nest). The plate was then sealed with transparent foil (Titer-Tops) and incubated for 20 h, at 37 °C with shaking. Following incubation, OD at 600 nm was measured using the Tecan Sunrise plate reader. Growth inhibition was determined by comparing the given sample with untreated culture (Growth Control—GC) with MHB alone (Sterility Control—SC) as an additional reference. The experiment was performed in at least two biological replicates of two technical replicates each. Statistical significance was determined by a Two-way ANOVA test using GraphPad Prism 9.

The minimal bactericidal concentration (MBC) values were determined, by diluting wells from the MIC experiment plate in fresh MHB medium. Dilutions of 10, 100, and 1000 times were prepared for the MIC well and up to two higher than MIC concentrations of the tested compound. Dilutions were made on a transparent flat-bottom 96-well plate (Nest) that was next sealed and incubated for 24 h, at 37 °C with shaking. Growth in a given sample well was compared with GC and SC controls. A particular concentration of a compound was considered bactericidal if no growth was observed for at least 100- and 1000-fold dilutions.

### Hemolytic activity

The hemolytic activity of the peptides against intact erythrocytes was tested using fresh red sheep blood erythrocytes (RBC). The 200 µL of red sheep blood was washed three times with the phosphate-buffered saline (PBS), 10 mM, pH 7.4 at 3500 rpm for 5 min. Then the cells were diluted in 10 mL of the PBS buffer, divided into 50 of 1.5 mL tubes, and pelleted by centrifugation. Each peptide concentration (200 µL) prepared in PBS (64, 32, 16, 8, 4, 2 µM) was added to red blood cells and incubated for 30 min (165 rpm, 37 °C), and then centrifuged (3500 rpm, 5 min). Next, 100 µL of supernatant from each tube was collected into a clear 96-well plate. The sample absorbance was measured at 405 nm using a spectrophotometer (Microplate Reader Biotek, Winooski, VT, United States). The hemolysis percentages were determined as

$$Hemolysis=\frac{\left(A-{A}_{0}\right)}{\left({A}_{100}-{A}_{0}\right)}\times 100$$

where *A*_*0*_ is the absorbance intensity of the RBC in the buffer (background), *A* is the absorbance intensity of RBC in the presence of peptides and A_100_ in the presence of Triton X-100. The erythrocyte suspension treated with 1% Triton X-100 (A_100_) was used as a positive control and the untreated suspension (A_0_) as a negative control.

## Results and discussion

### Synthesis and design of the stapled (KFF)_3_K analogs

**Figure 1:**
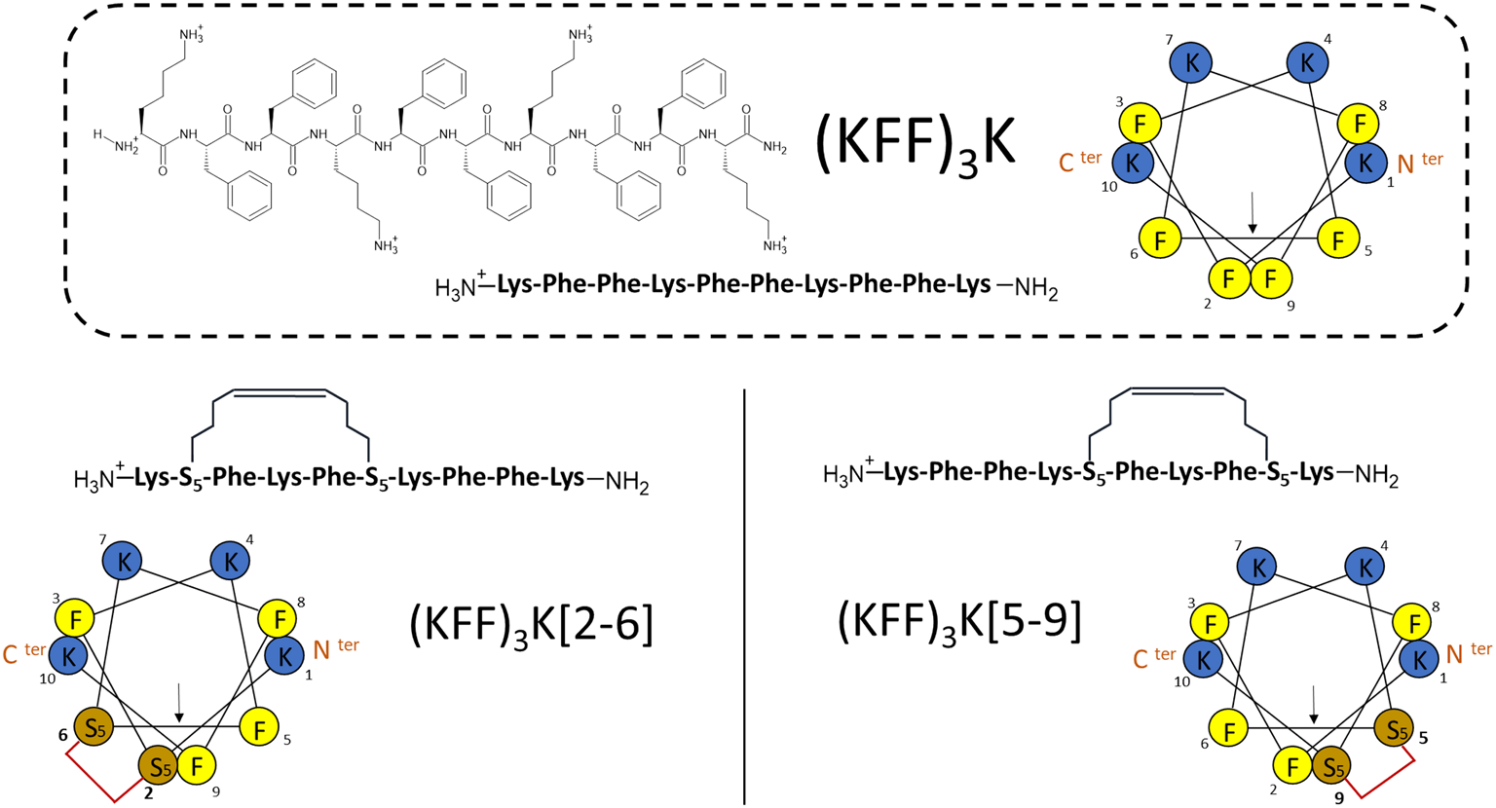
Chemical structure of (KFF)_3_K and the sequence of the stapled peptides (KFF)_3_K[2–6] and (KFF)_3_K[5–9]). The helical projection of the peptides and positions of the staples are highlighted. All peptides were amidated at the C-terminus for biostability^72^.

We synthesized two variants of the stapled (KFF)_3_K peptide: (KFF)_3_K[5–9] and (KFF)_3_K[2–6] (Fig. 1). To preserve the amphipathic nature of (KFF)_3_K, the unnatural amino acid always replaced Phe. We have previously shown that replacing a hydrophobic amino acid (Leu or Ile) and adding a hydrocarbon staple in the anoplin sequence increased its antimicrobial activity^47^. Therefore, in (KFF)_3_K, we selected the staple sites to initiate the active secondary structure that could trigger the destructive effect on the bacterial cell membrane. The peptide amino acid sequence is symmetric, so the two stapled peptides differ in the directionality of the backbone secondary structure. Peptides were synthesized by manual peptide synthesis on a resin (see “Materials and methods”) and purified by HPLC (Supplementary Figs. S1–S3). Hydrocarbon stapling involves the incorporation of two non-natural amino acids with olefinic side chains: (*S*)-2-(4′-pentenyl)-alanine (further denoted PEN) during solid-phase synthesis. These residues were located at positions separated by one helical turn (*i* and *i* + 4)^39^. Before the peptide cleavage from the resin, the olefinic groups were cross-linked by ruthenium-catalyzed ring-closing metathesis^44^.

### Secondary structure of the (KFF)_3_K peptides

**Figure 2:**
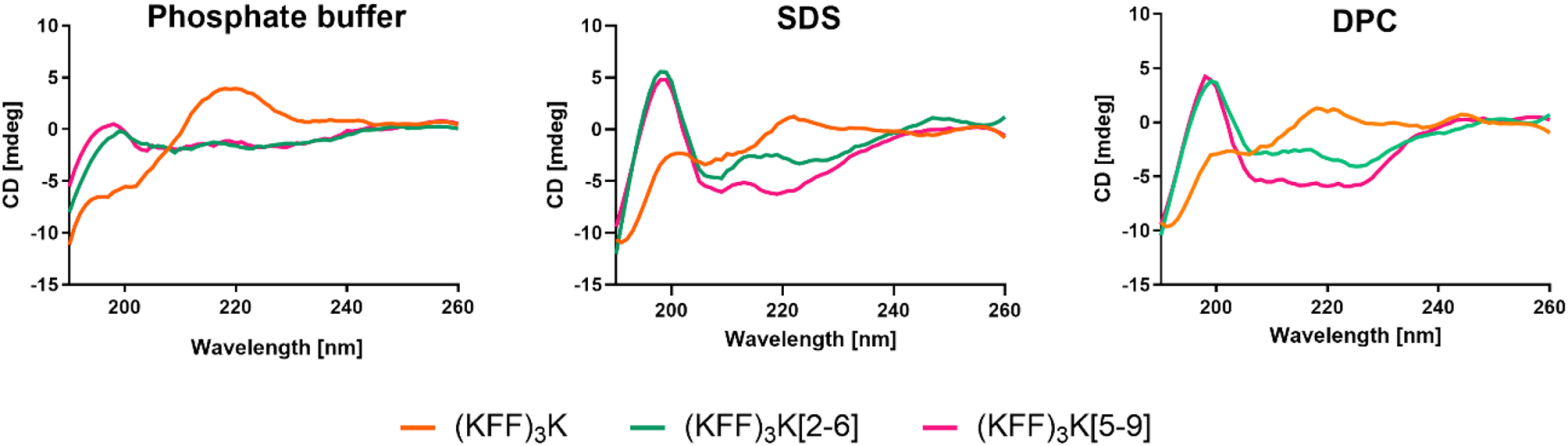
CD spectra obtained for (KFF)_3_K, (KFF)_3_K[2–6] and (KFF)_3_K[5–9] in the phosphate buffer (10 mM, pH 7.0), SDS (5 mM) and DPC (2 mM) micelles.

To determine the secondary structures of the peptides in the phosphate buffer and micellar environment of sodium dodecyl sulfate (SDS) and dodecylphosphocholine (DPC), we used circular dichroism (CD) spectroscopy. The spectra shown in Fig. 2 confirm that (KFF)_3_K in the buffer solution is predominantly unstructured, which agrees with our previous studies of the unstapled (KFF)_3_K^19^. However, introducing the hydrocarbon staple changed the peptide spectra indicating somewhat stabilized secondary structures already in the buffer. The appearance of minima in the 205–225 nm region indicates that the peptide structure changed from an unstructured random coil to a more helical one. In addition, for the stapled forms, the percentage of helicity and β-sheet calculated using DichroWeb^50^ (Table 1) increased.

In the presence of micelles, the spectra of the stapled (KFF)_3_K[2–6] and ((KFF)_3_K[5–9] show two characteristic minima at 208 and 222 nm, indicating a helical structure (Fig. 2). The spectrum for (KFF)_3_K[5–9] has deeper minima with a ratio of R = 1.01 (R = [Θ]_222_/[Θ]_208_), indicating a stable ⍺-helix structure, whereas for (KFF)_3_K[2–6] the ratio is weaker (R = 0.69), suggesting the 3_10_-helix fragments^73^. In contrast, the unstapled (KFF)_3_K in SDS is disordered. The SDS micelles are negatively charged and commonly used as the simplest mimics of bacterial membranes. The applied micelle concentrations were above the critical micelle concentration (CMC) for SDS (CMC = 4.5 mM) and DPC (CMC = 1.1 mM)^74,75^. In the DPC solution, the CD spectra indicate that all peptides adopt a helical structure (Fig. 2, Table 1), with (KFF)_3_K[5–9] showing the highest helicity of about 60%.

Nevertheless, from the CD spectra alone we cannot conclude about the secondary structure of (KFF)_3_K in micelles apart from the tendency towards a helix. We also increased the concentrations of SDS to 10 mM and DPC to 5 mM and observed that (KFF)_3_K forms a clear helix in the DPC solution. The spectra in 10 mM SDS are similar to those at 5 mM (Supplementary Fig. S4). For the stapled analogues, both spectral bands also indicate the ⍺-helical conformation.

### Structural dynamics of the (KFF)_3_K peptides

**Figure 3:**
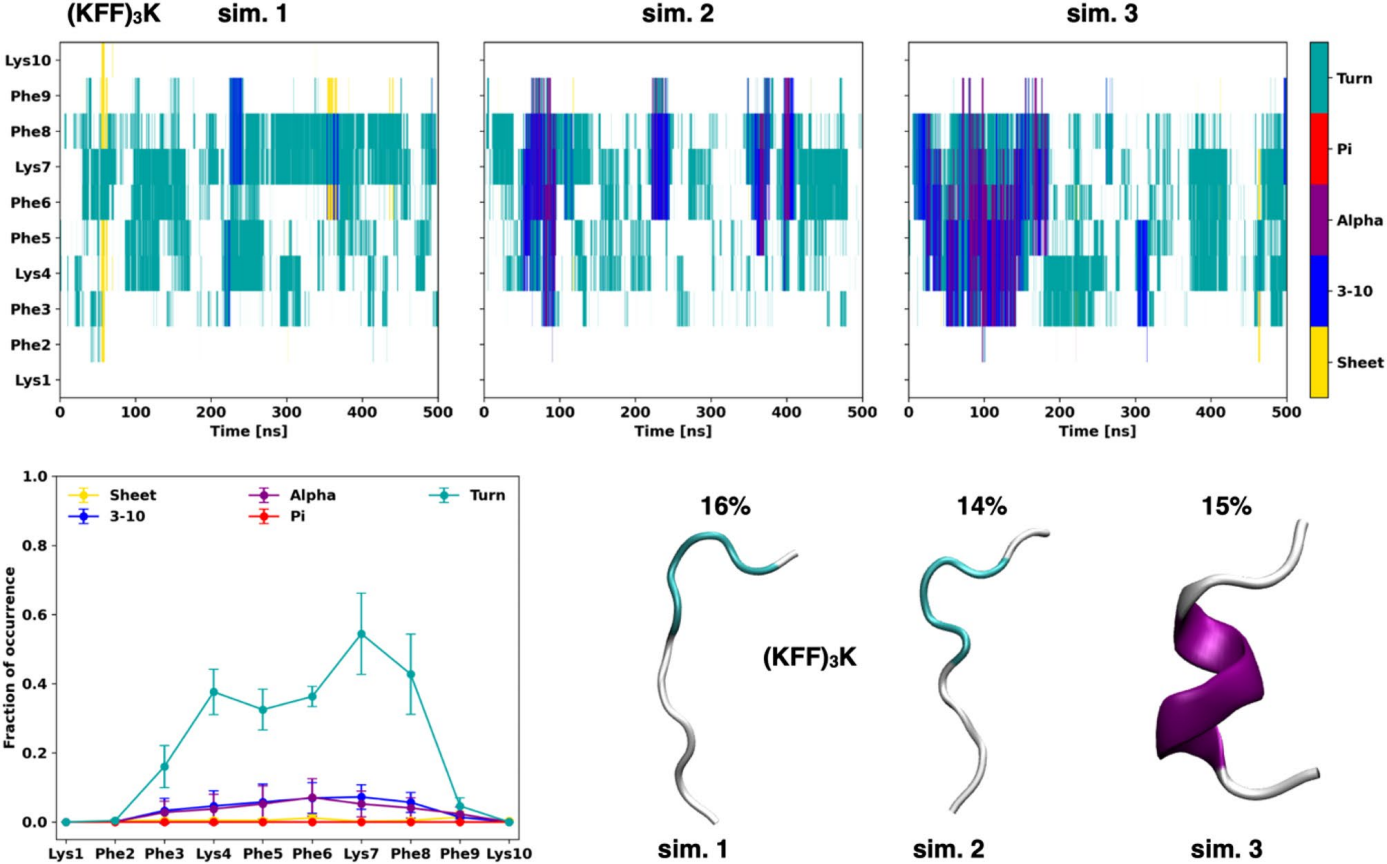
Top: The secondary structure changes as a function of the simulation time from three atomistic MD simulations of the (KFF)_3_K peptide in explicit solvent. The peptide starting conformation was unstructured. Bottom: The fraction of occurrence of the secondary structure type for each amino acid and the most populated cluster representative (with occupancy) from each simulation.

To investigate the tertiary structure and conformational dynamics of the (KFF)_3_K analogues, we further performed atomistic molecular dynamics (MD) simulations. For the unmodified (KFF)_3_K in the simulation conditions mimicking the phosphate buffer (see “Materials and methods”), regardless of the peptide starting conformation, the (KFF)_3_K did not show one dominant structure and was mainly unstructured. The secondary structure of (KFF)_3_K as a function of the simulation time is shown in Fig. 3 (starting from the unstructured form) and Supplementary Fig. S5 (starting from the helical form). In some simulations, we observed a preference to adopt or keep either the 3_10_- or ⍺-helix. Although the helices were transient on a 100–200 ns scale, their appearance indicates that the peptide can form a helical structure in the buffer solution, which corroborates the DichroWeb analyses (Table 1). Detection of the 3_10_-helix in this short peptide corroborates the fact that such helices form most often at the N- or C-termini and can also transition into an ⍺-helix.

**Figure 4:**
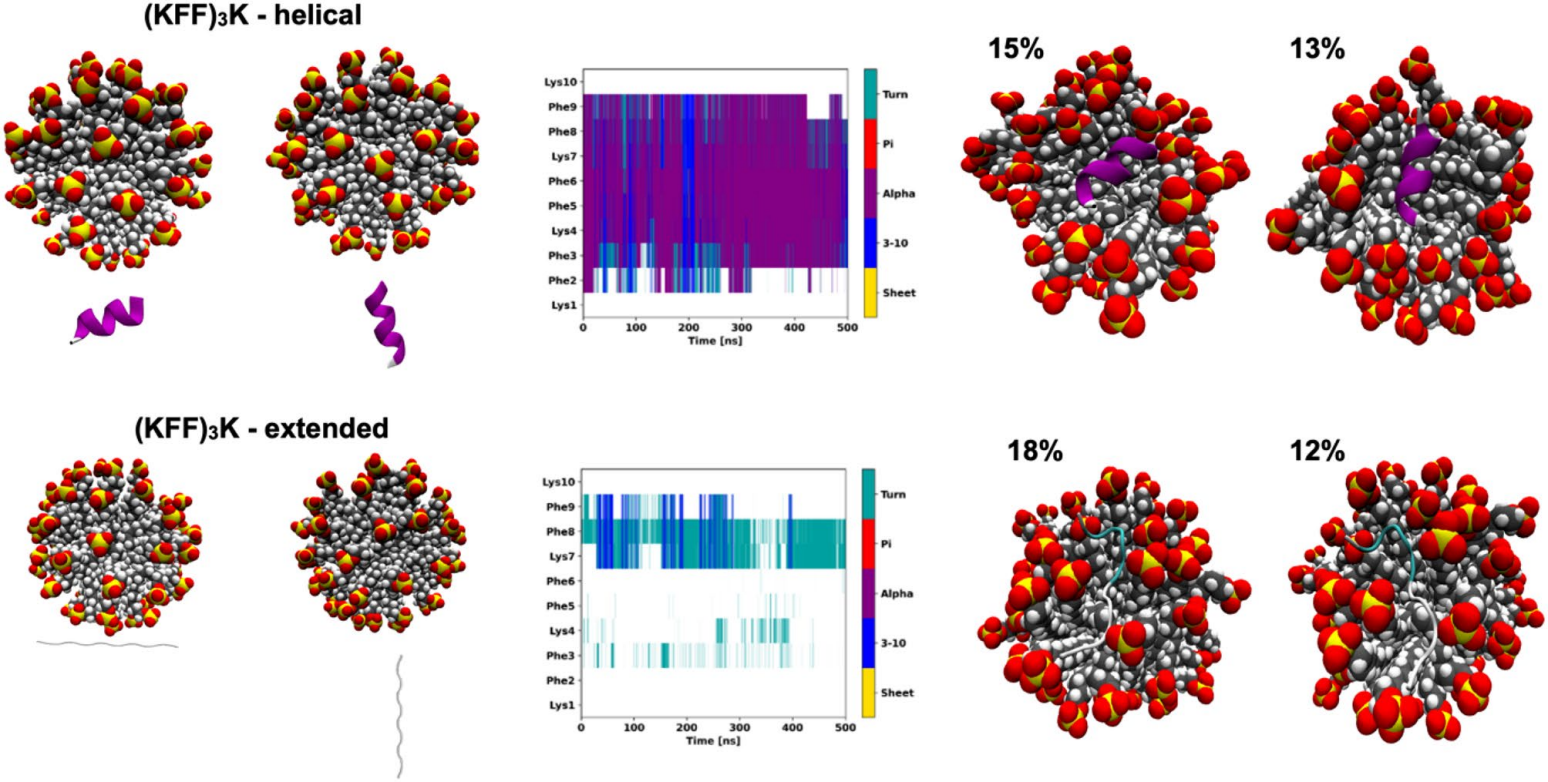
The secondary structure changes as a function of the simulation time from atomistic MD simulations of the (KFF)_3_K peptide in the presence of the SDS micelle in explicit solvent. The starting conformations either in helical or extended forms (left) and the two most populated cluster representatives with their occupancies (right) are also shown.

The unmodified (KFF)_3_K peptide positioned in a helical conformation near SDS interacts and penetrates the surface of this micelle (Fig. 4). The (KFF)_3_K peptide keeps the starting helical form in all MD simulations confirming that the micelle prevents the helix from unfolding. On the other hand, in the simulations starting from the unstructured (KFF)_3_K next to SDS, the peptide does not fold into a stable helix, but the timescale of the simulations might be too short to observe this event. However, as evidenced by CD spectroscopy, (KFF)_3_K in the presence of SDS is similarly structurally diverse as in the phosphate buffer, and CD does not show helix stabilization in the unstapled peptide (Fig. 2).

### Structural dynamics of the modified (KFF)_3_K

The (KFF)_3_K[2–6] and (KFF)_3_K[5–9] peptides simulated in the buffer from stapled but extended conformations did not form helices. Although in one, out of three simulations, for both peptides we observed a 3_10_-helix (Supplementary Fig. S6), it lasted for only 50–100 ns. This may be because the peptide backbone was constrained by the covalent stapling. However, similar simulations starting from the helical forms confirmed that the staple can stabilize an ⍺-helix, although stabilization occurred mainly within the sequence covered by the staple and the end regions fluctuated (Supplementary Fig. S7).

Therefore, we simulated the peptides modified with the PEN residues, (KFF)_3_K(2,6) and (KFF)_3_K(5,9), but not stapled, which allowed for PEN flexibility. For both modified peptides, we observed helix formation (Supplementary Fig. S8). Interestingly, similar helical structures were observed in CD spectroscopy for anoplin that was modified with PEN residues but not stapled^47^. This confirmed that the replacement of Phe by PEN enhanced helix formation in these peptides, especially in the fragment between the PEN residues. So indeed, the staple may hinder the ability of the backbone to twist. On the other hand, the helical conformations with distances between the PEN residues of about 5 Å suggest that the PEN side chains can adopt the geometry necessary to form a covalent bond, in accord with the *i*, *i* + 4 position in the sequence corresponding to one helical turn (Supplementary Fig. S8).

**Figure 5:**
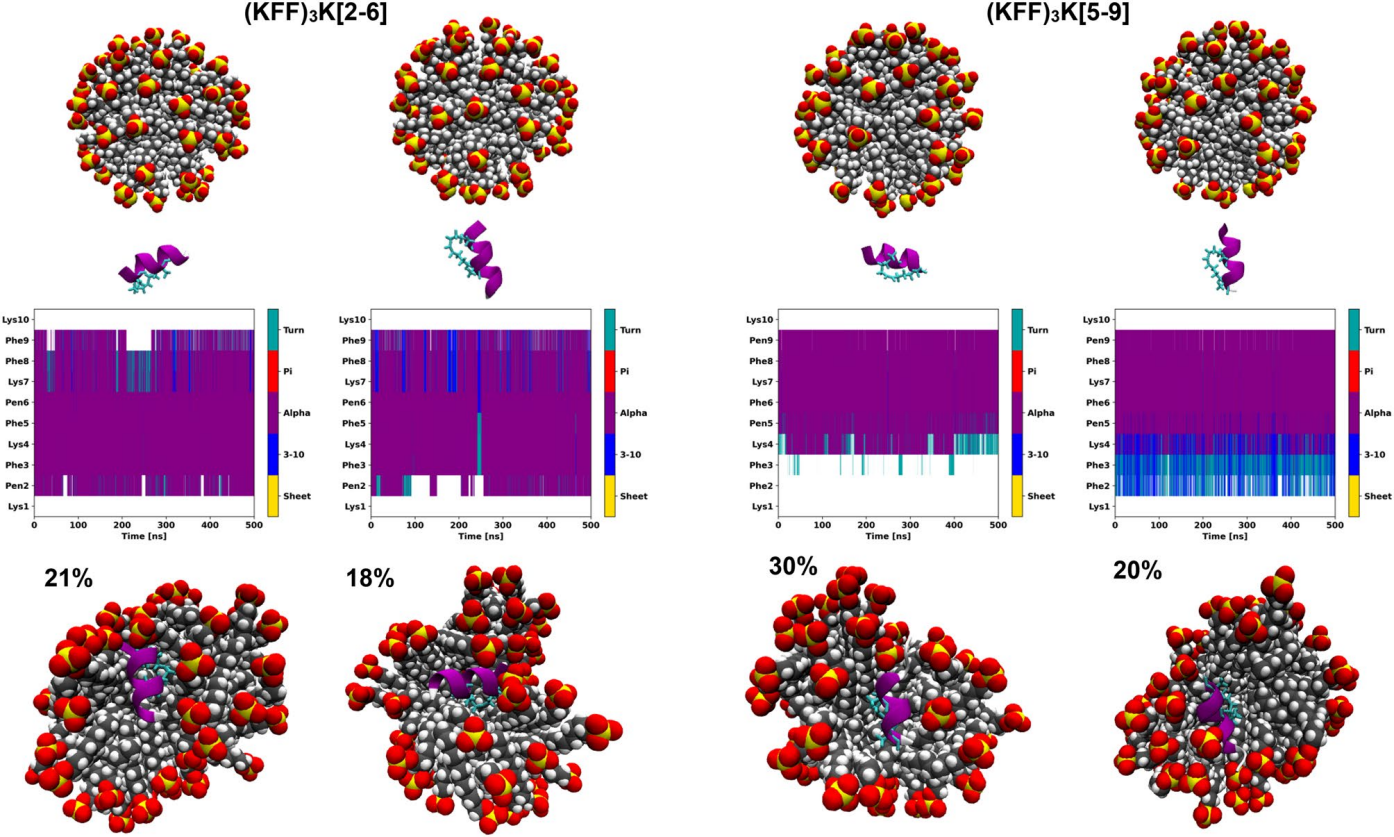
The secondary structure changes as a function of the simulation time from atomistic MD simulations of the stapled (KFF)_3_K peptides and SDS micelle in explicit solvent. The starting structures are shown in the top panels and the two most populated cluster representatives (with occupancies) are in the bottom panels.

Based on the above conformations of (KFF)_3_K(2,6) and (KFF)_3_K(5,9), we constructed the stapled peptides and simulated them near the SDS micelle. Both stapled peptides kept their helical forms and penetrated the SDS micelle (Fig. 5). Again, as expected, in the simulations starting from extended conformations, the stapled peptides did not form helices, except a stable 3_10_-helix at the N-terminus in the simulation of the (KFF)_3_K[5–9] (Supplementary Fig. S9).

MD simulations suggest that the structural dynamics of the helix depends on the location of the staple. For (KFF)_3_K[2–6], we observed a stable helix covering the entire sequence, with a few events where the ⍺-helix converted to the 3_10_-helix at the C-terminus (Fig. 5). The (KFF)_3_K[5–9] peptide showed an unstructured N-terminus (or with unstable 3_10_-helix) and a helix formed from the C-terminus^76^. This could explain the previously discussed deeper CD spectra for the (KFF)_3_K[5–9] (Fig. 2).

### Stability of peptides in the solution of α-chymotrypsin

**Figure 6:**
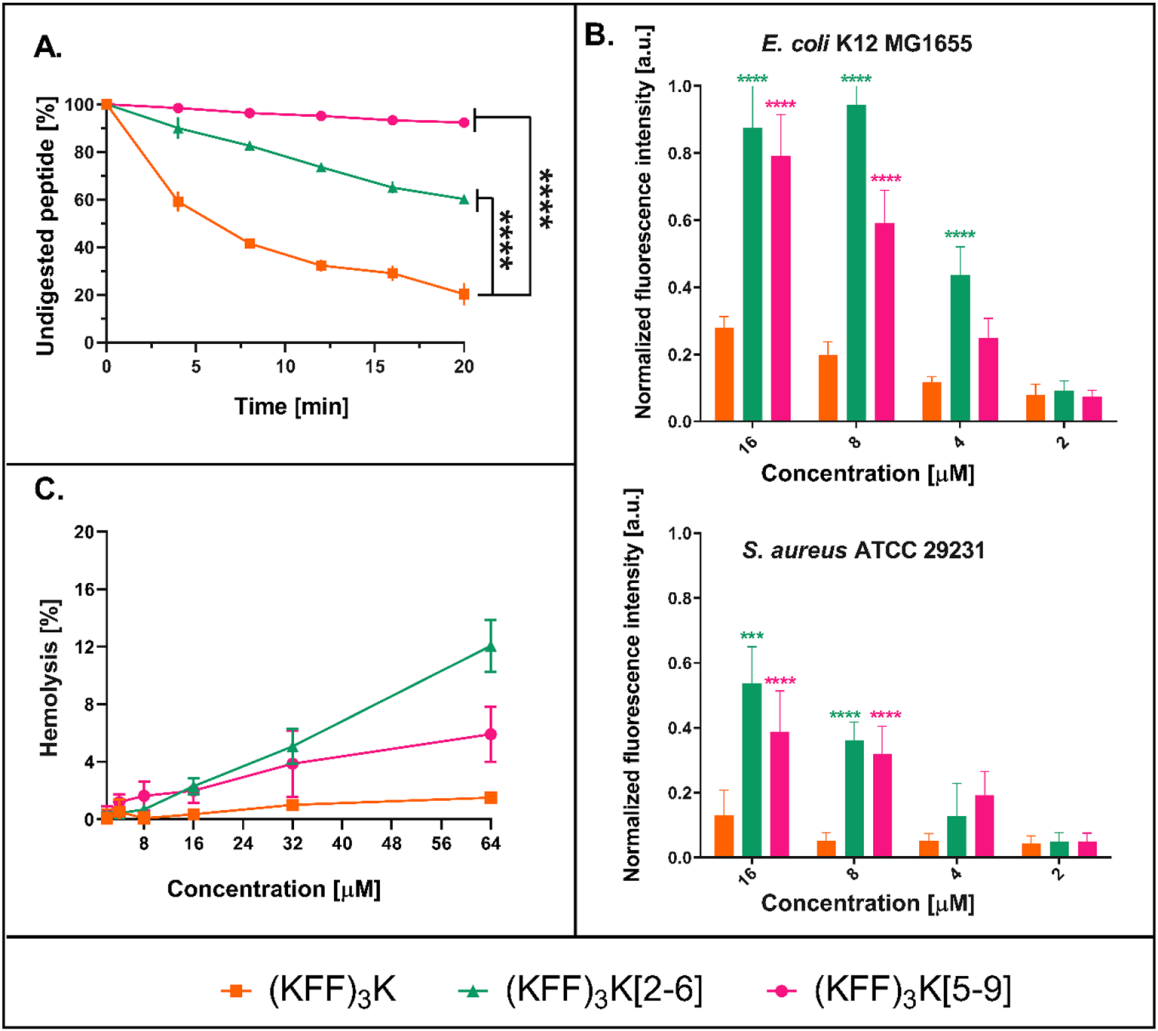
(**A**) Digestion of peptides by α-chymotrypsin at indicated times. The percentage of the digested peptide was determined by the area under the HPLC peak. (**B**) Permeabilization assay of *E. coli* K12 MG1655 and *S. aureus* ATCC 29213 using propidium iodide (PI) shown as normalized fluorescence intensity after 30 min of incubation with the peptide. Error bars represent the standard error of the mean; n = 3 (**C**) Hemolytic activity of the (KFF)_3_K analogs against the sheep erythrocytes incubated with varying concentrations of the peptide for 30 min. Erythrocytes treated with 1% Triton-X-100 were used as a positive (100% of hemolysis) control. Error bars represent the standard error of the mean; n = 3. Statistical significance between the (KFF)_3_K and the stapled peptides (for **A** and **B**), (KFF)_3_K[2–6] and (KFF)_3_K[5–9], is denoted by ****p < 0.0001, ***p < 0.001. Otherwise, the differences are not statistically significant.

We investigated the stability of the (KFF)_3_K peptide and its stapled analogs in the presence of the α-chymotrypsin enzyme (Fig. 6A), which hydrolyzes the peptide bond at the carboxyl side of aromatic amino acids such as Phe^77^. Since in the (KFF)_3_K sequence, the modified amino acids (PEN) were inserted instead of Phe, α-chymotrypsin was chosen as a model protease to test how these modifications affect the stability of the (KFF)_3_K analogs.

The effects of protease digestion were determined by HPLC. The increasing number and frequency of signals appearing within the main peak over time indicate low stability of (KFF)_3_K (Supplementary Fig. S10). After 20 min of incubation with chymotrypsin, 70% of the (KFF)_3_K peptide was degraded. The stapled (KFF)_3_K[5–9] and (KFF)_3_K[2–6] exhibited much higher stability against chymotrypsin than (KFF)_3_K. This is confirmed by the reduced number of signals appearing at the HPLC chromatogram during time analysis (Supplementary Fig. S10). As hypothesized, incorporating PEN at positions 2, 6 and 5, 9 of (KFF)_3_K and further stapling reduced the number of potential cleavage sites for chymotrypsin from 6 to 2.

The proteolytic digestion shown as the percentage of peptide degradation in chymotrypsin solution (Fig. 6A) illustrates that (KFF)_3_K[2–6] and, especially, (KFF)_3_K[5–9] are more stable than unmodified (KFF)_3_K. The higher stability of (KFF)_3_K[5–9] relative to (KFF)_3_K[2–6] may be due to the location of the staple which, in the latter, is closer to the N-terminus. The N- and C-termini add asymmetry to the peptide in terms of the staple positions (Fig. 1). Overall, hydrocarbon stapling proved to significantly increase (KFF)_3_K stability in accord with previous literature for other peptides^78^.

### Bacterial membrane degradation by the (KFF)_3_K analogues

Using the PI uptake assay, we evaluated whether the modified (KFF)_3_K exhibited antibacterial character by destabilizing the membrane of Gram-negative *E. coli* K12 MG1655 and Gram-positive *S. aureus* ATCC 29213 strains. PI is a fluorescent dye that cannot cross the bacterial cell membrane on its own. When the continuity of the membrane is disrupted, the dye enters the cell and binds to bacterial DNA resulting in an increase in fluorescence that can be monitored^71^.

We found that the unstapled (KFF)_3_K does not disrupt membrane continuity, which corroborates the fact that it is a CPP (Fig. 6B). Even if (KFF)_3_K adopts a secondary structure close to the membrane, it is most probably transient and does not disintegrate the membrane enough for the PI to reach the cellular interior. (KFF)_3_K[2–6] shows a slightly better permeabilizing effect on the bacterial cell membrane than (KFF)_3_K[5–9]. Against the *E. coli* K12 strain, the former achieves a permeabilization efficiency of 0.8–0.9 of normalized fluorescence intensity at concentrations of 8–16 µM, whereas (KFF)_3_K[5–9] of up to 0.75. These peptides are also able to permeabilize Gram-positive bacteria with a thick cell wall, which hinders their interaction with the membrane^79^. This explains the lower fluorescence intensity after 30 min of incubation compared to Gram-negative bacteria. Again, (KFF)_3_K[2–6] is more effective at permeabilizing the cell wall of Gram-positive bacteria.

Overall, the stapled (KFF)_3_K analogues allow for higher PI uptake than (KFF)_3_K, suggesting increased bacterial cell envelope destabilization. The PI assay demonstrated that the (KFF)_3_K peptides stabilized with a hydrocarbon staple act by destabilizing the bacterial cell membrane, according to the mechanism of action of a typical AMP.

### Antibacterial and hemolytic activity

To test the antibacterial activity of the stapled (KFF)_3_K analogs, we determined their MIC and MBC against several Gram-positive and Gram-negative bacterial strains, including both non-pathogenic laboratory and pathogenic bacterial strains carrying resistance to various antibiotics (Table 2, Supplementary Figs. S11–S16). The antibiotic polymyxin B was selected as the reference because of its similarities in both the structure (it is a cyclic peptide) and mode of action (disturbance of the cell membrane) to the (KFF)_3_K. The compounds were tested at concentrations up to 32 µM, the lowest observed MIC for the (KFF)_3_K alone.

**Table 2:** The MIC and MBC of various (KFF)_3_K peptide forms compared with polymyxin B.

Strain	MIC and MBC (µM)							
	(KFF)_3_K		(KFF)_3_K[2–6]		(KFF)_3_K[5–9]		polymyxin B	
	MIC	MBC	MIC	MBC	MIC	MBC	MIC	MBC
*E. coli* O157:H7 ST2-8624 (Clinically-derived, pathogenic strain^80^*)*	32	≥ 32	4	4	8	8	0.5–1	0.5–1
*E. coli* 1841-06 (β-lactam resistant, clinically-derived strain^81^*)*	> 32	> 32	4	4	4	8	1	1
*S. aureus* ATCC 29213 (Preceptrol strain^82^)	> 32	> 32	16	16–32	8–16	16	> 32	> 32
*S. aureus* ATCC BAA1720 MRSA (methicillin-resistant, clinically-derived strain^83^)	> 32	> 32	8	16–32	16	32	> 32	> 32
*Ps. aeruginosa* ATCC 27853 (Preceptrol strain^84^)	32	≥ 32	4–8	8	4	4	1	1
*E. coli* K12 (reference, non-pathogenic strain^85^)	32	32	2	2	2	2	0.125	0.125

As expected, polymyxin B showed high efficiency against Gram-negative strains with MIC ranging between 0.25 and 1 µM (Table 2), coherent with the literature values for *E. coli* strains^86,87^. Contrary, polymyxin B was not active against Gram-positive strains, probably because of the thick peptidoglycan layer preventing it from reaching the plasma membrane. The non-stapled (KFF)_3_K, did not inhibit the growth of any strain at concentrations up to 32 µM, in accord with its classification as a CPP. Conversely, both stapled (KFF)_3_K inhibited the growth of all Gram-positive and Gram-negative strains, with MIC ranging between 2 and 16 µM. Interestingly, both stapling patterns resulted in comparable MIC within a given strain, which indicates that the secondary structure of the stapled peptides was the main contributor (and not the position of the staple with respect to the N- or C-terminus).

Polymyxin is a last-resort antibiotic for treating multidrug-resistant Gram-negative pathogens^88^. Although the MIC obtained for the stapled (KFF)_3_K peptides in Gram-negative strains were higher than those for polymyxin B, they fit within the concentration ranges that could still be considered useful in clinical applications, especially if we consider numerous recent reports showing the spread of resistance to polymyxins^88^. What is more, the activity of the stapled (KFF)_3_K is mostly bactericidal, and not only bacteriostatic, as witnessed by almost identical MIC and MBC values (Table 2).

In addition to their decent antibacterial properties, the (KFF)_3_K analogs show low hemolytic activity. The (KFF)_3_K peptide shows negligible hemolysis of sheep red blood cells even at concentrations as high as 64 µM (Fig. 6C). In turn, (KFF)_3_K[5–9] and (KFF)_3_K[2–6] exhibit hemolytic activity between 4 and 12% at 64 µM. This agrees with the peptides showing more helicity (Table 1), especially in the DPC medium. Indeed, the stapled peptides can be more hemolytic than their natural forms^47^, because of different hydrophobicity and stabilization of the secondary structure. Importantly, the stapled (KFF)_3_K peptides are practically not hemolytic at concentrations at which they inhibited bacterial growth (2–32 µM).

## Conclusions

Based on the sequence of the cell-penetrating peptide (KFF)_3_K, we designed and synthesized its hydrocarbon stapled analogues, (KFF)_3_K[2–6] and (KFF)_3_K[5–9]. Modifications were introduced, symmetrically to the N- and C- terminus, at two Phe positions, not to change the peptide net charge and amphipathicity. The unmodified (KFF)_3_K was not active against any Gram-positive or Gram-negative strains at concentrations up to 32 µM, in accord with its classification as a CPP. However, (KFF)_3_K stapling at both positions ([2–6] and [5–9]) decreased the MIC against both Gram-negative and Gram-positive bacteria at least two- and up to 16-fold. This proved that stapling of (KFF)_3_K initiated its antibacterial activity. Interestingly, within a given strain, MIC were comparable for both stapling patterns, which indicates that not the position of the staple but the adopted secondary structure was the crucial factor. Further, we showed that both stapled (KFF)_3_K destabilize the bacterial membrane, following the mechanism of action of a typical AMP. However, (KFF)_3_K[2–6] permeabilized the envelope of Gram-positive and Gram-negative bacteria slightly better than (KFF)_3_K[5–9], as evidenced by the PI assay. In addition, we found that hydrocarbon stapling improved peptide stability in the chymotrypsin solution, as the substitution of Phe removed the potential hydrolysis sites for this enzyme. CD spectra and MD simulations indicated that (KFF)_3_K does not adopt a dominant secondary structure in the buffer, whereas helical content increases to about 40% in the presence of SDS. The stapled (KFF)_3_K[2–6] and KFF_3_K[5–9] show over 50% of helical content in the micellar environment, which seems crucial for their antibacterial activity. MD simulations suggest that the stapled peptides penetrate the SDS micelle but the helical content depends on the position of the staple. A key aspect of using membrane-active peptides as antimicrobial agents is to avoid adverse effects on eukaryotic cells. Indeed, in hemolytic assays, the stapled peptides showed low hemolytic activity at concentrations at which they inhibited bacterial growth. In conclusion, stapling of the (KFF)_3_K peptide converts this CPP peptide into an AMP. Indeed, many CPP can act as AMP and vice versa, many AMP may possess cell-penetrating properties^89–91^. For this reason, we cannot establish that the stapled (KFF)_3_K analogs are only antimicrobial because distinguishing between AMP and CPP is tenuous and depends on the peptide concentration and membrane properties^1^. Nonetheless, to the best of our knowledge, this is the first work that uses the hydrocarbon stapling technique to initiate the antibacterial activity of the CPP peptide (KFF)_3_K. Our results suggest that this is an approach that can be used in the future to design and obtain compounds with better antimicrobial and perhaps even therapeutic applications.

### Supplementary Information

Supplementary Figures.

## Data Availability

The data generated or analyzed during this study are available from the corresponding author upon reasonable request.
