# Supplementary material for: Structural dynamics influences the antibacterial activity of a cell-penetrating peptide (KFF)3K
Source: Sci Rep. 2023 Sep 8;13:14826. doi: 10.1038/s41598-023-38745-y (PMC10491836; doi:10.1038/s41598-023-38745-y)
Supplement: Supplementary file 1 — Supplementary Figures. [file 41598_2023_38745_MOESM1_ESM.pdf]

Supplementary material

Structural dynamics influences the antibacterial activity of a cell-penetrating peptide $(KFF)_3K$

Julia Macyszyn[1], Piotr Chyży[1], Michał Burmistrz[1], Małgorzata Lobka[1,2], Joanna Miszkiewicz[1,3], Monika Wojciechowska[1], Joanna Trylska[1]*

[1]Centre of New Technologies, University of Warsaw, Warsaw, Poland
[2]Faculty of Physics, University of Warsaw, Warsaw, Poland
[3]College of Inter-Faculty Individual Studies in Mathematics and Natural Sciences, University of Warsaw, Warsaw, Poland

*** Correspondence:**
Joanna Trylska, joanna@cent.uw.edu.pl

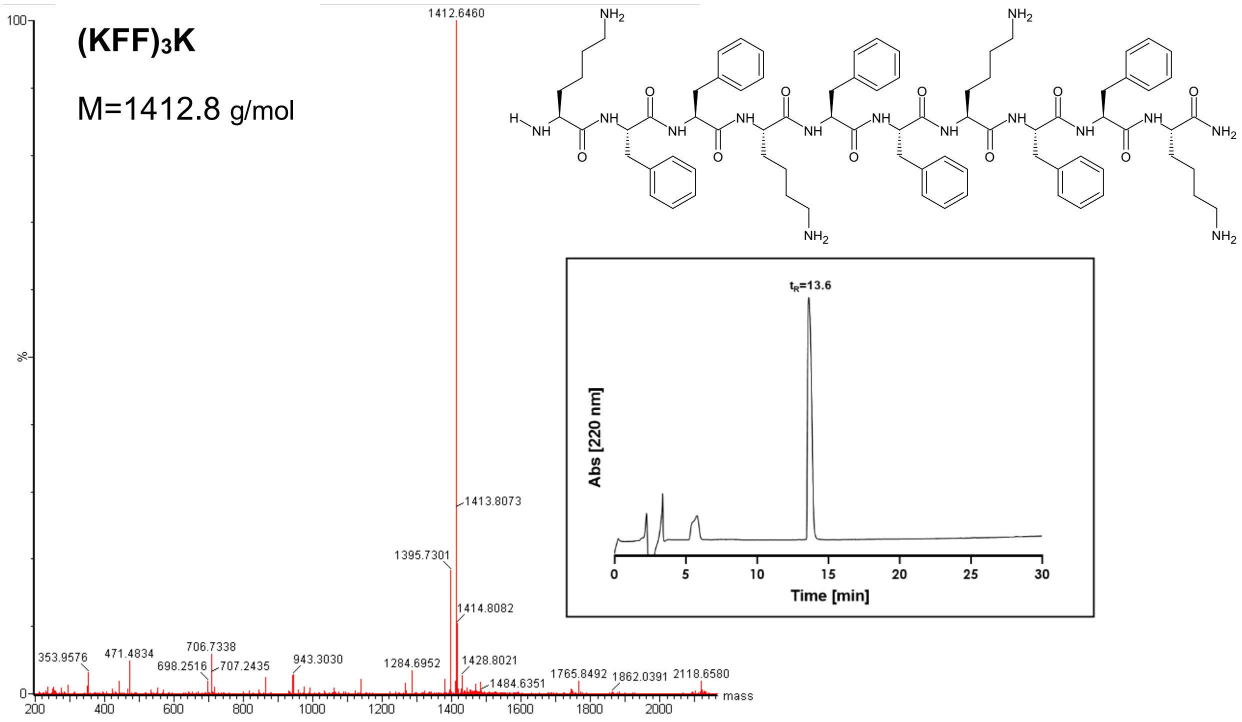

Supplementary Figure S1. The mass spectrum, chemical structure, calculated mass and RP-HPLC chromatogram of $(KFF)_3K$.

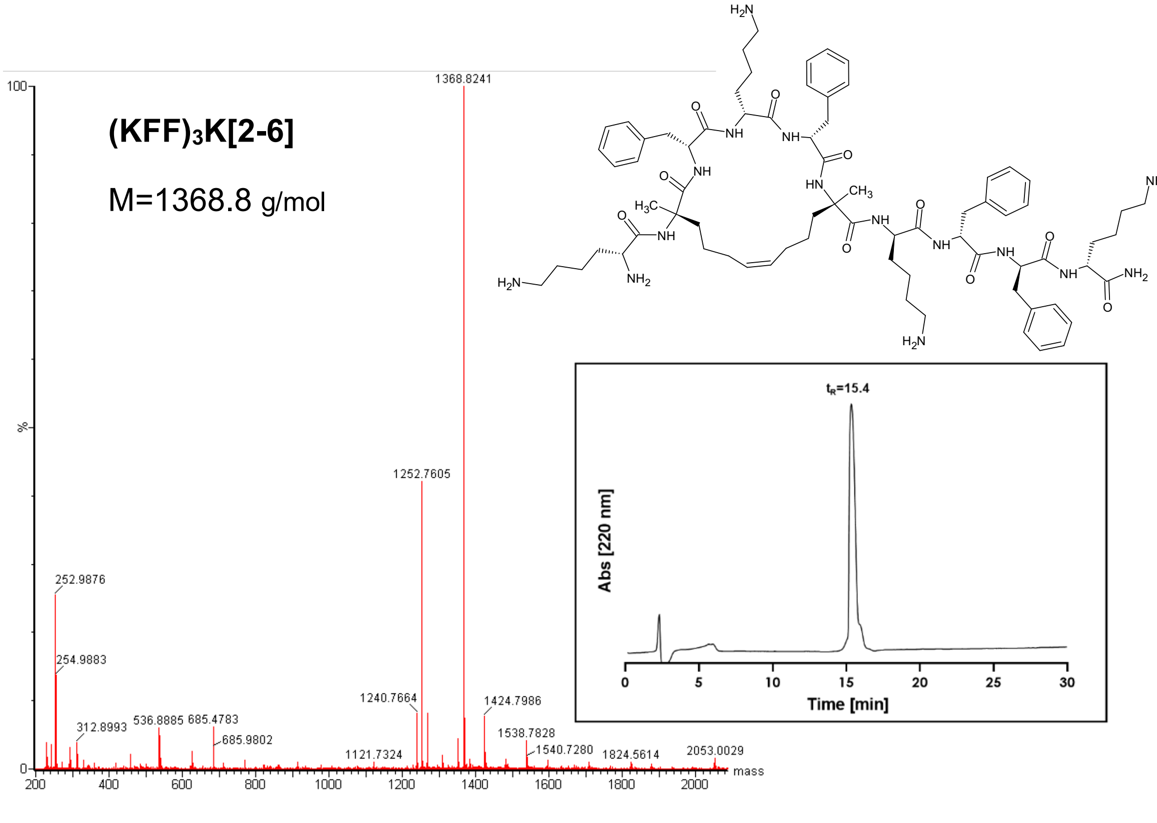

Supplementary Figure S2. The mass spectrum, chemical structure, calculated mass and RP-HPLC chromatogram of $(KFF)_3K[2\text{-}6]$.

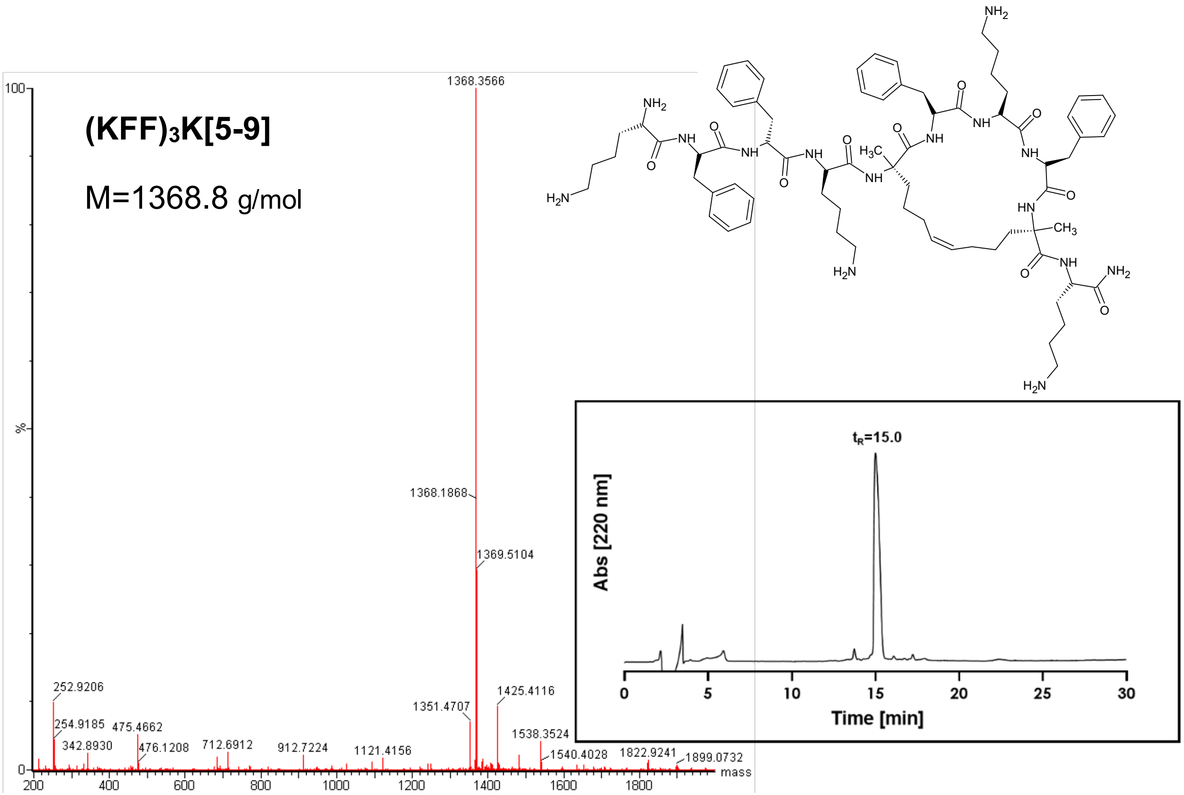

Supplementary Figure S3. The mass spectrum, chemical structure, calculated mass and RP-HPLC chromatogram of (KFF)$_3$K[5-9].

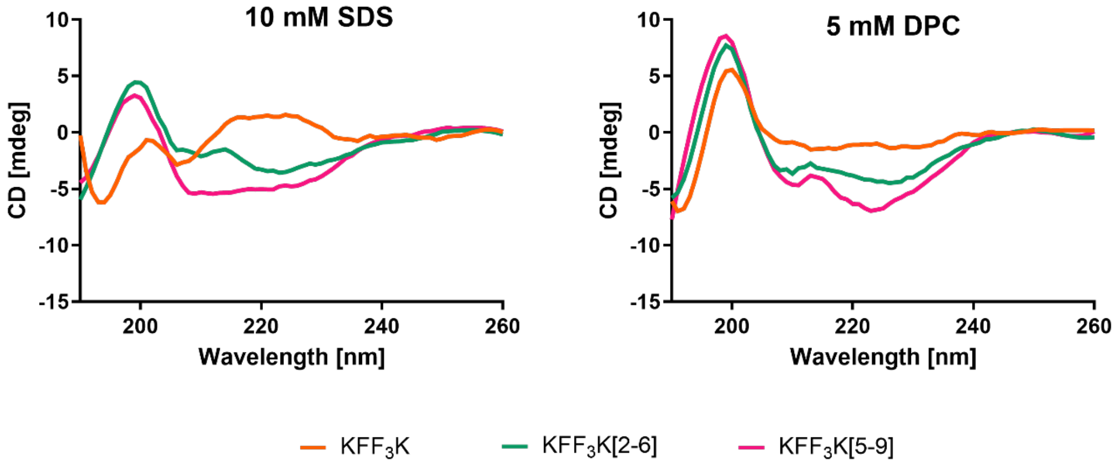

Supplementary Figure S4. CD spectra obtained for (KFF)$_3$K, (KFF)$_3$K[2-6] and (KFF)$_3$K[5-9] in the increased concentrations of micelle environment: SDS (10 mM) and DPC (5 mM).

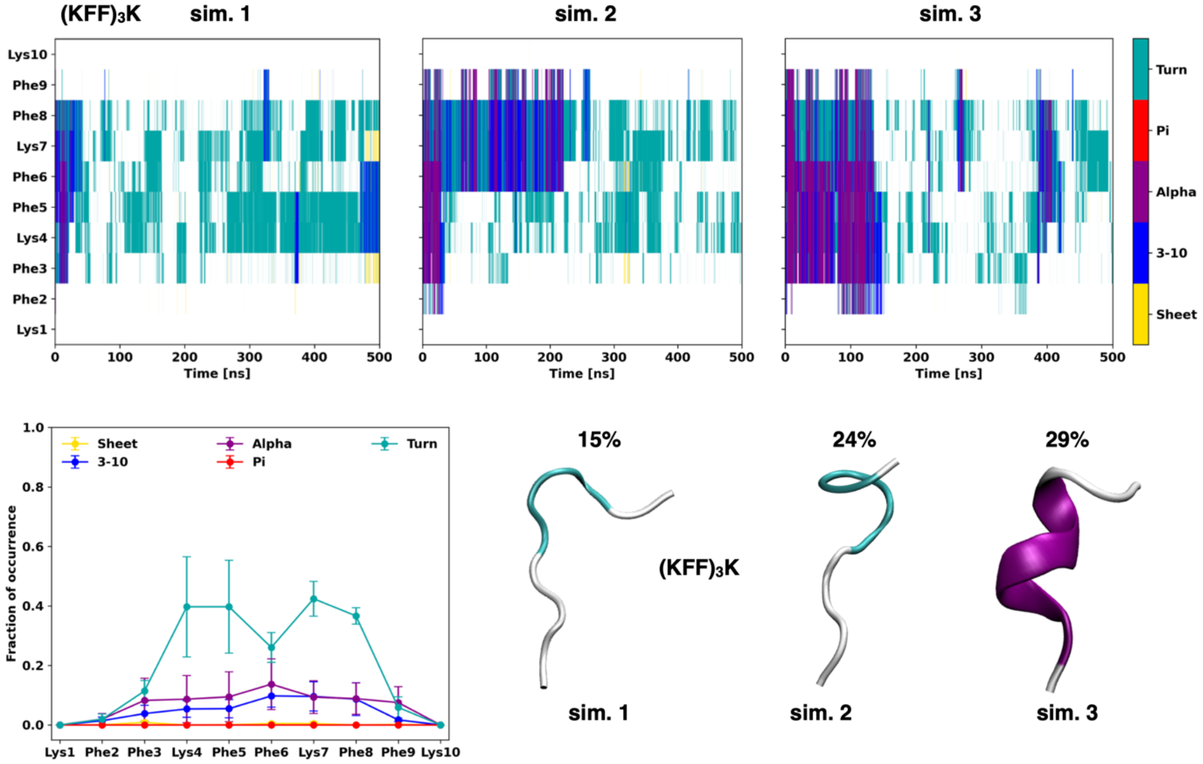

Supplementary Figure S5. Top: The secondary structure changes as a function of the simulation time from three atomistic MD simulations of the $(KFF)_3K$ peptide in explicit solvent. The starting structure of the peptide was in helical form. Bottom: The fraction of occurrence of secondary structure type for each amino acid and the most populated cluster representative (with occupancy) from each simulation.

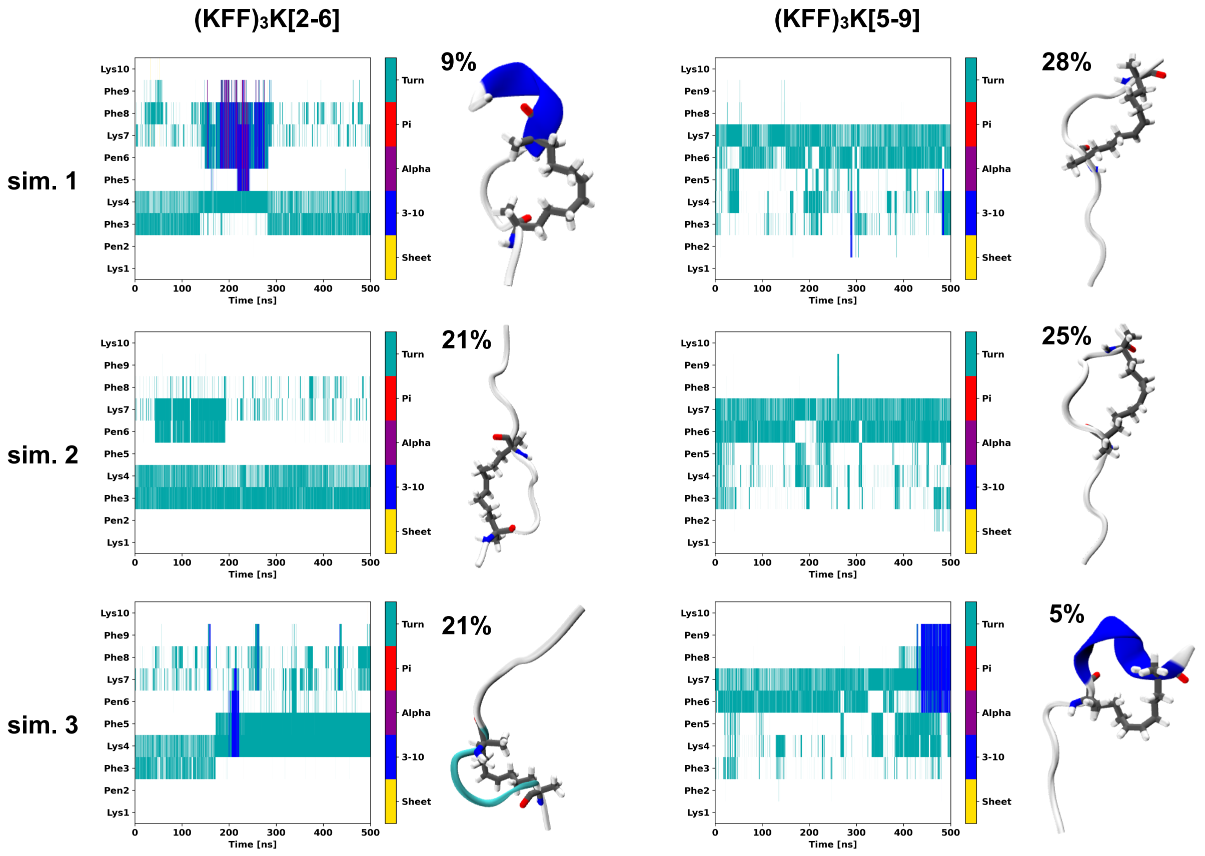

Supplementary Figure S6. The secondary structure changes as a function of the simulation time from three atomistic MD simulations of the stapled (KFF)$_3$K peptides in explicit solvent, with disordered starting structures. The most populated cluster representatives (with occupancies) from each simulation are also shown. The backbone direction corresponds to labels in the X-axes of the graphs, with the N-termini in the bottom and C-termini at the top. Pen labels the stapled residues.

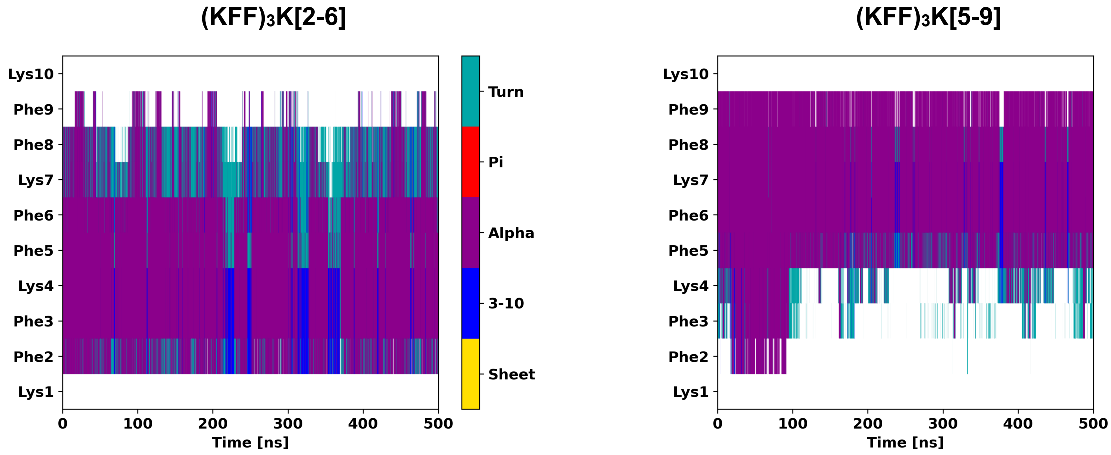

Supplementary Figure S7. The secondary structure changes as a function of the simulation time from atomistic MD simulations of the stapled (KFF)$_3$K peptides in explicit solvent. The starting structures of the peptides were in a helical form.

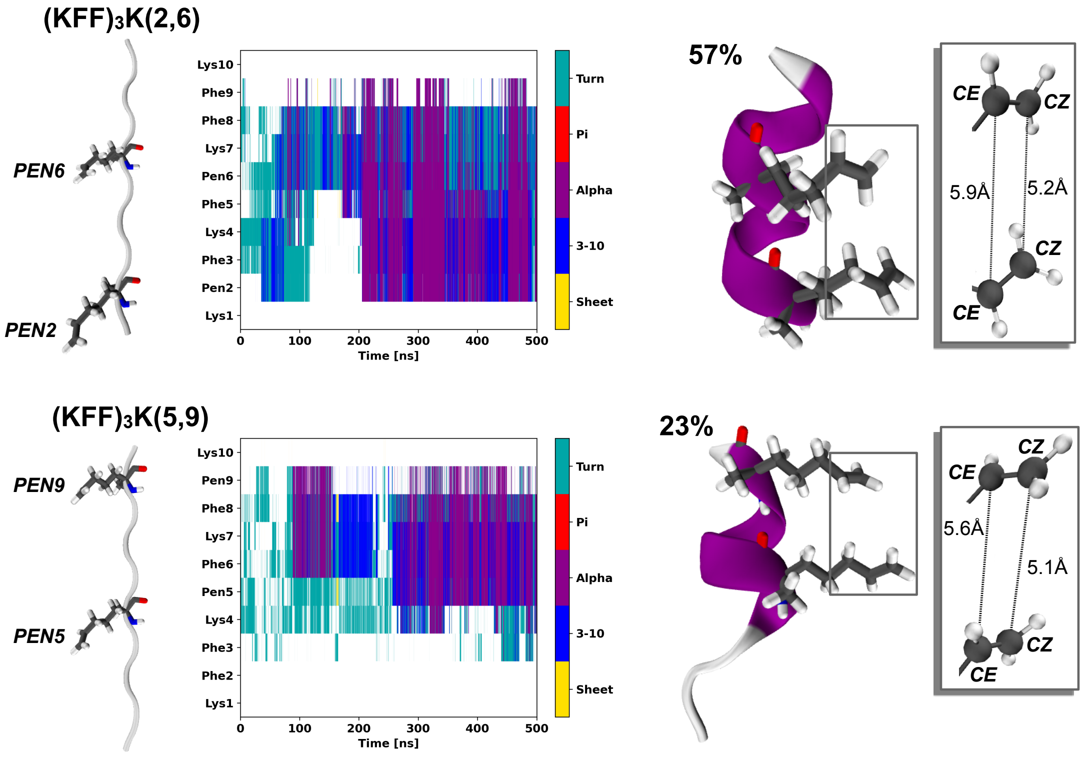

Supplementary Figure S8. The formation of helical structures shown as a function of the simulation time from atomistic MD simulations of the $(KFF)_3K$ peptides with two Phe replaced with PEN residues (2-(4'-pentenyl)-alanine) in explicit solvent. The starting structures of the peptides are shown on the left and the most populated clusters (with occupancies) on the right.

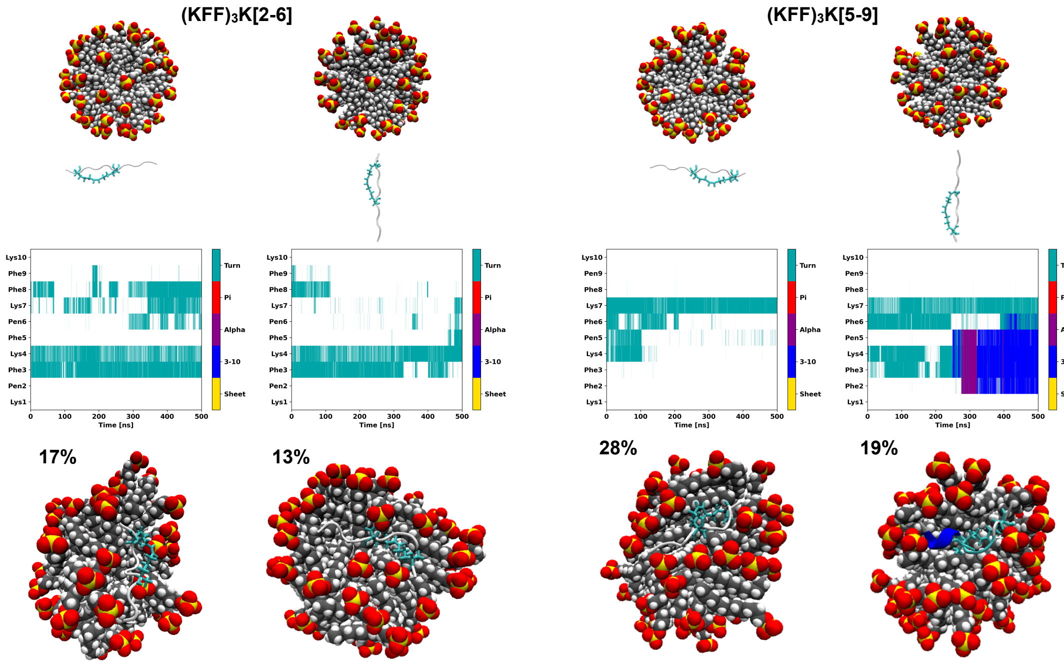

Supplementary Figure S9. The secondary structure changes as a function of the simulation time from atomistic MD simulations of the stapled $(KFF)_3K$ peptides near the SDS micelle in explicit solvent. The starting structures are shown in the top and the two most populated cluster representatives (with occupancies) in the bottom panels.

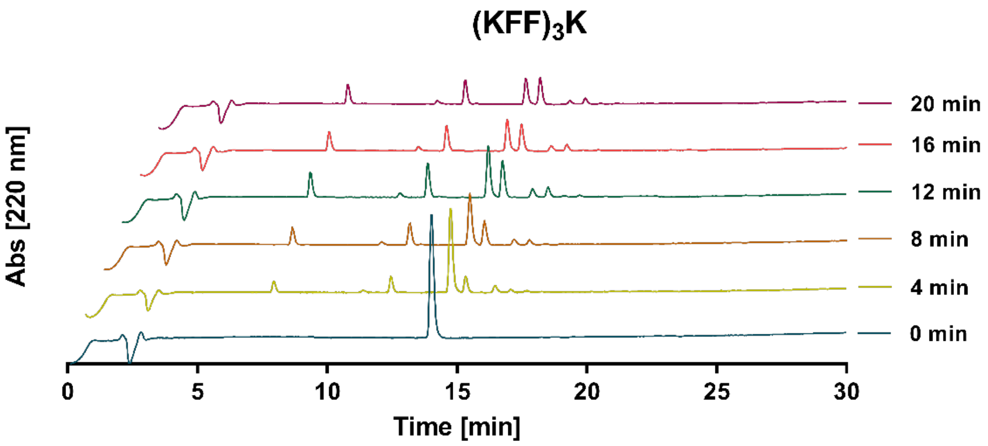

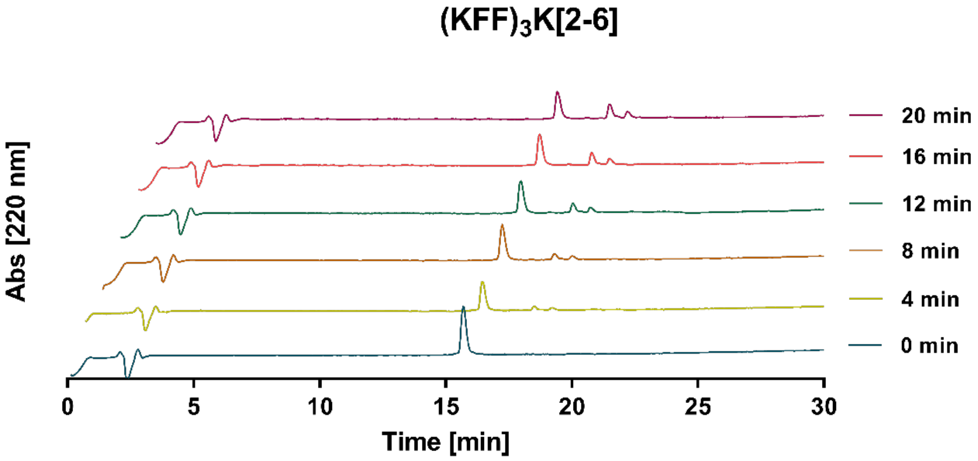

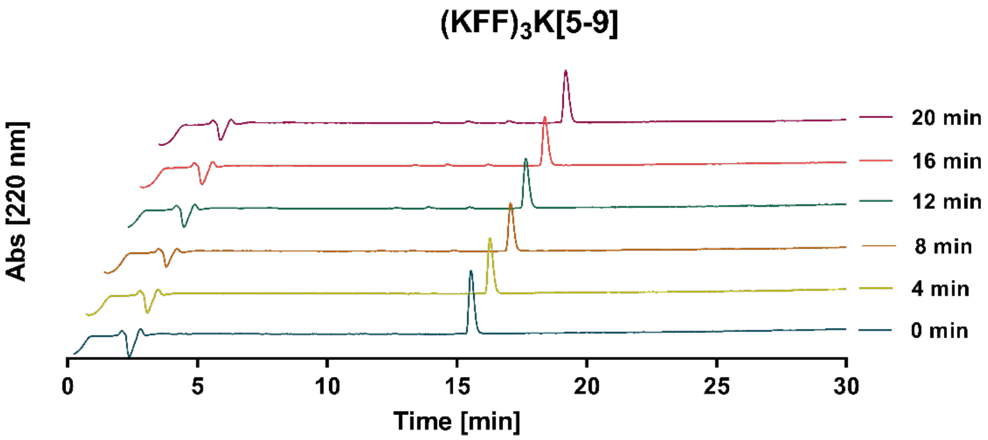

Supplementary Figure S10. The analytical RP-HPLC chromatograms (220 nm) of peptides $(KFF)_3K$, $(KFF)_3K$[2-6] and $(KFF)_3K$[5-9] at different time of incubation with α-chymotrypsin solution.

E. coli 1841-06

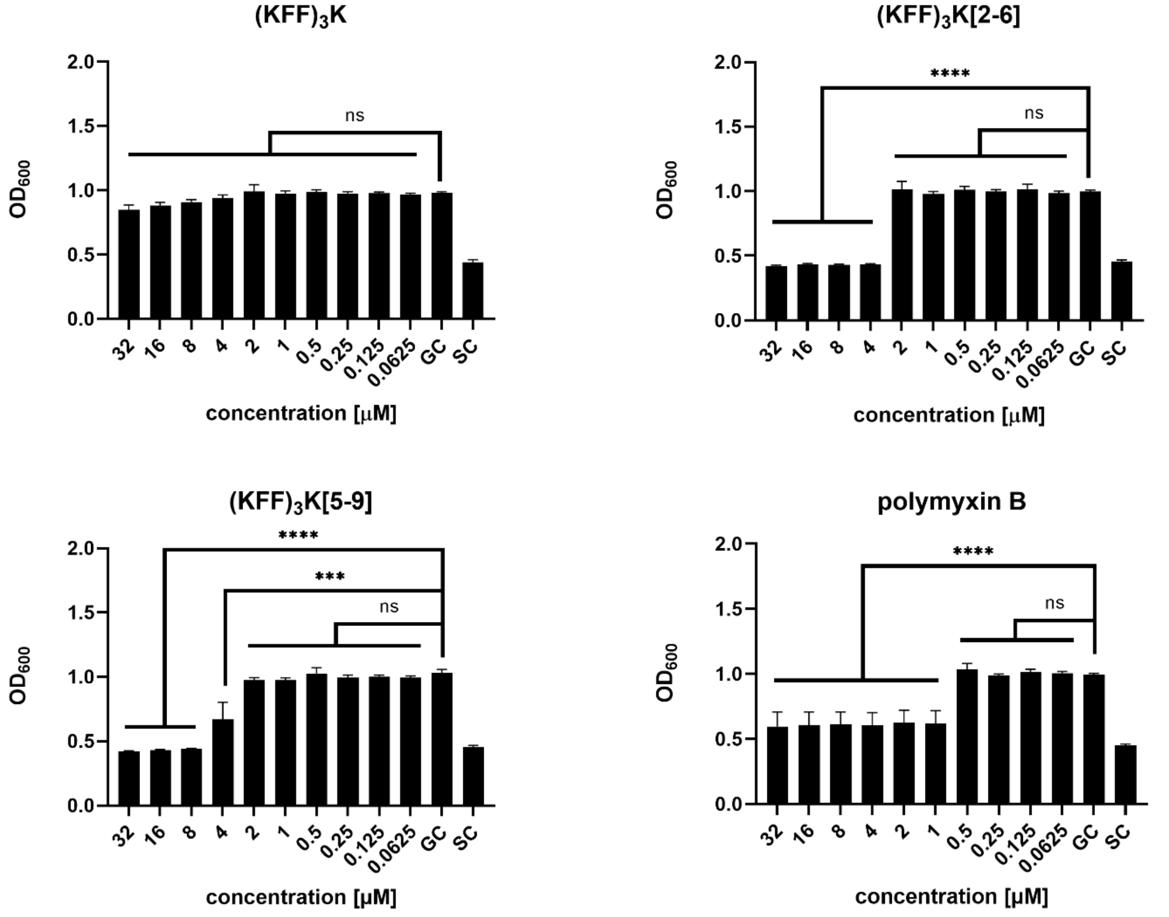

Supplementary Figure S11. The MIC results for the *E. coli* 1841-06 strain. Non-stapled (KFF)3K together with stapled peptides and polymyxin B were tested in various concentrations. GC – growth control, SC – sterility control. For each sample experiment was repeated in two biological replicates of two technical replicates each. Error bars represent the standard error of the mean. Statistical significance between the samples and GC: **** P < 0.0001, *** P < 0.001, ns – not significant.

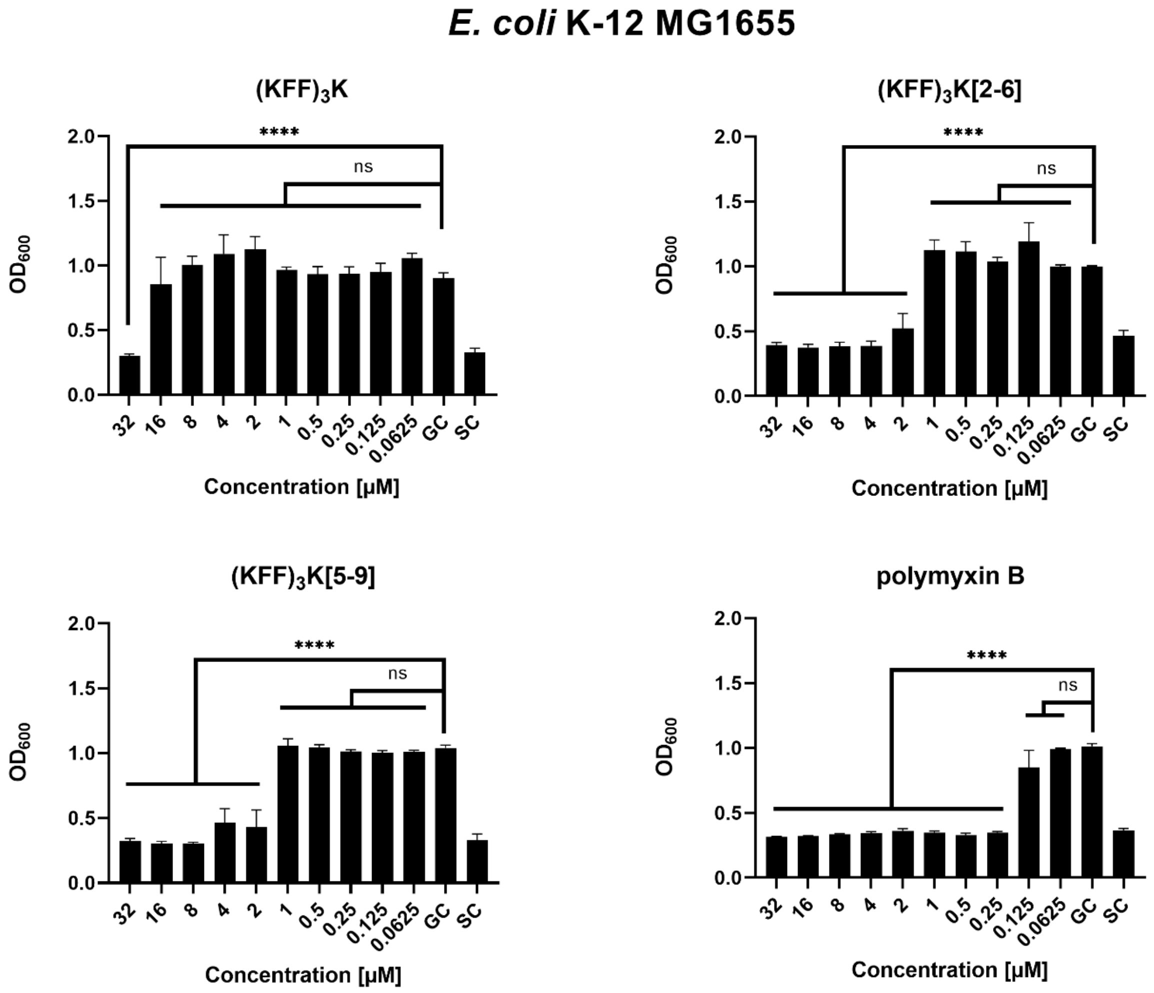

Supplementary Figure S12. The MIC results for the *E. coli* K-12 MG1655 strain. Non-stapled (KFF)$_3$K together with stapled peptides and polymyxin B were tested in various concentrations. GC – growth control, SC – sterility control. For each sample experiment was repeated in two biological replicates of two technical replicates each. Error bars represent the standard error of the mean. Statistical significance between the samples and GC: **** P < 0.0001, ns – not significant.

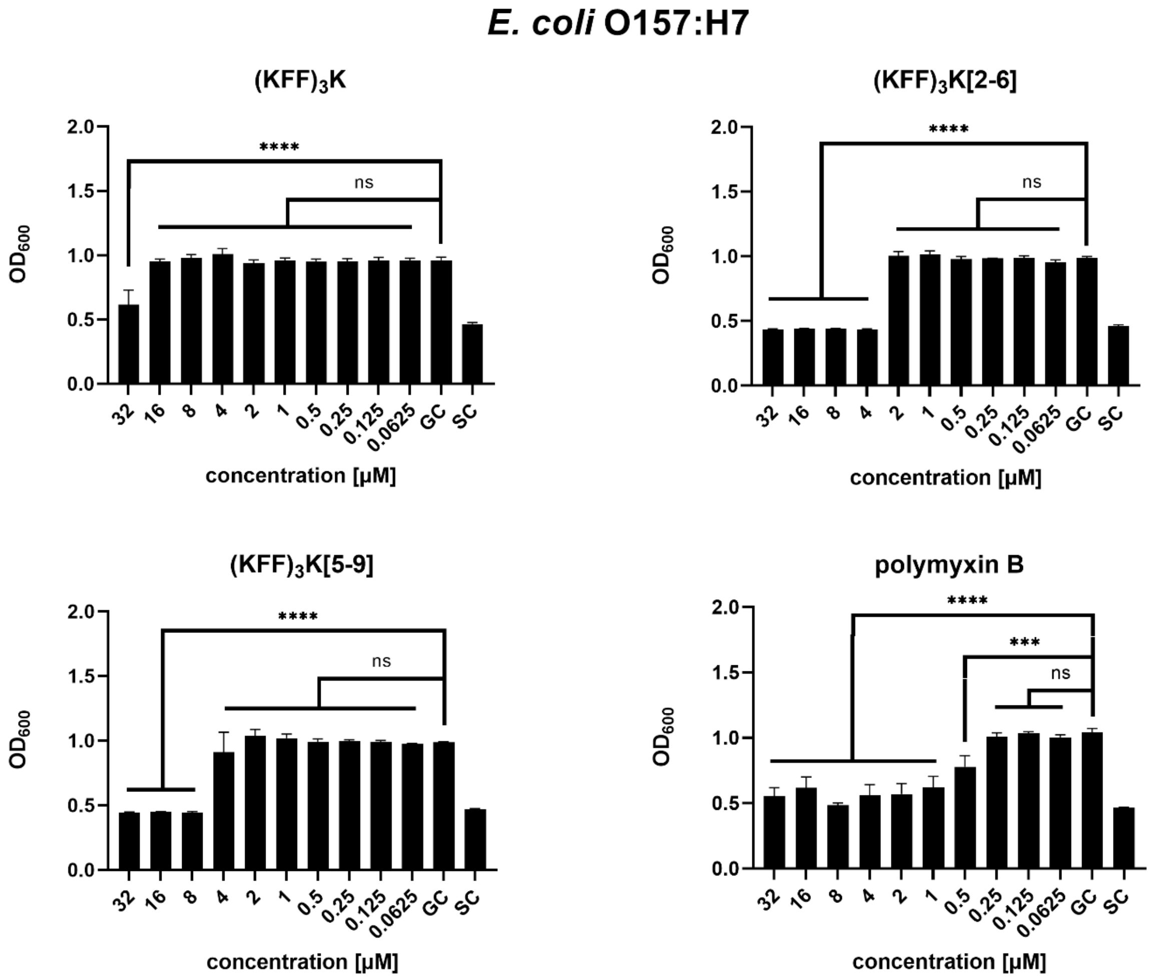

Supplementary Figure S13. The MIC results for the *E. coli* O157:H7 ST2-8624 strain. Non-stapled $(KFF)_3K$ together with stapled peptides and polymyxin B were tested in various concentrations. GC – growth control, SC – sterility control. For each sample experiment was repeated in two biological replicates of two technical replicates each. Error bars represent the standard error of the mean. Statistical significance between the samples and GC: **** P < 0.0001, *** P < 0.001, ns – not significant.

Ps. aeruginosa ATCC 27853

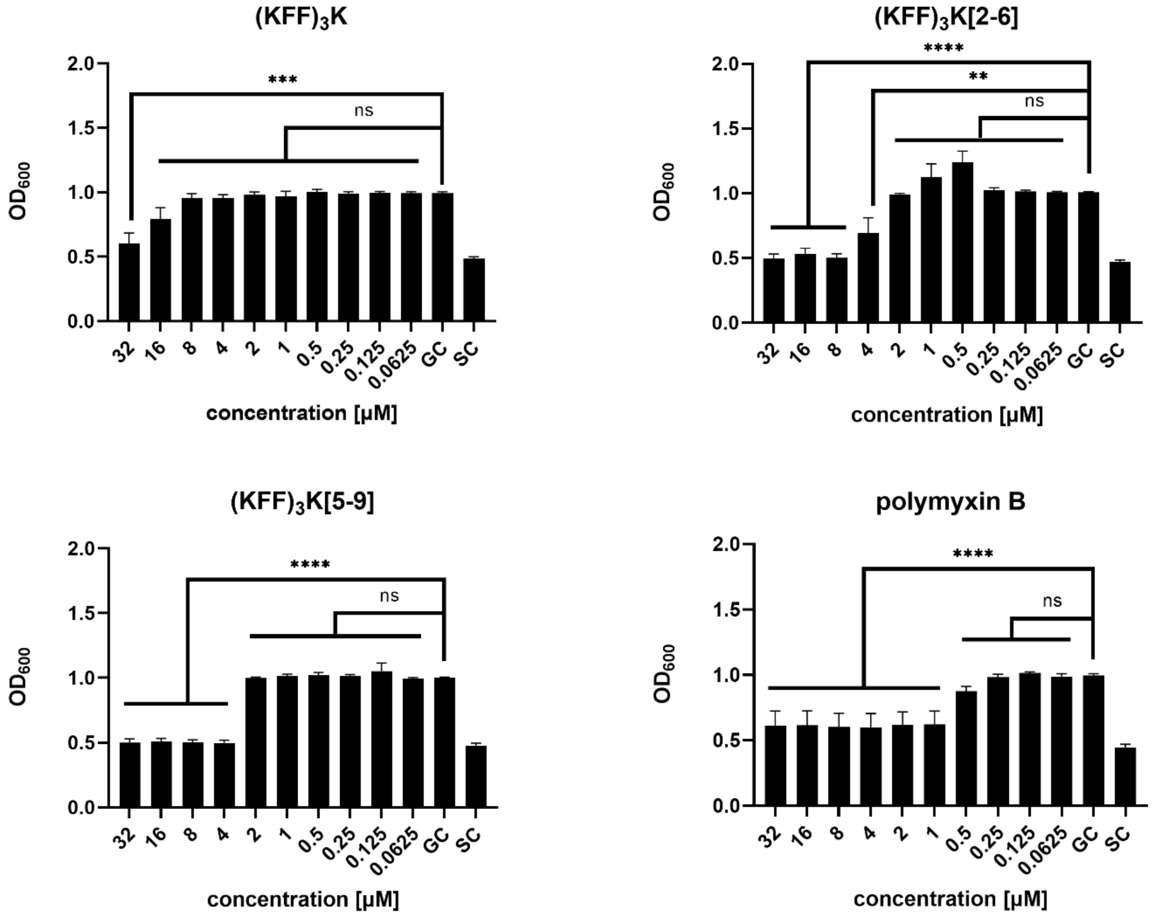

Supplementary Figure S14. The MIC results for the *Ps. aeruginosa* ATCC 27853 strain. Non-stapled (KFF)$_3$K together with stapled peptides and polymyxin B were tested in various concentrations. GC – growth control, SC – sterility control. For each sample experiment was repeated in two biological replicates of two technical replicates each. Error bars represent the standard error of the mean. Statistical significance between the samples and GC: **** $P < 0.0001$, *** $P < 0.001$, ** $P < 0.01$, ns – not significant.

***S. aureus* ATCC 29213**

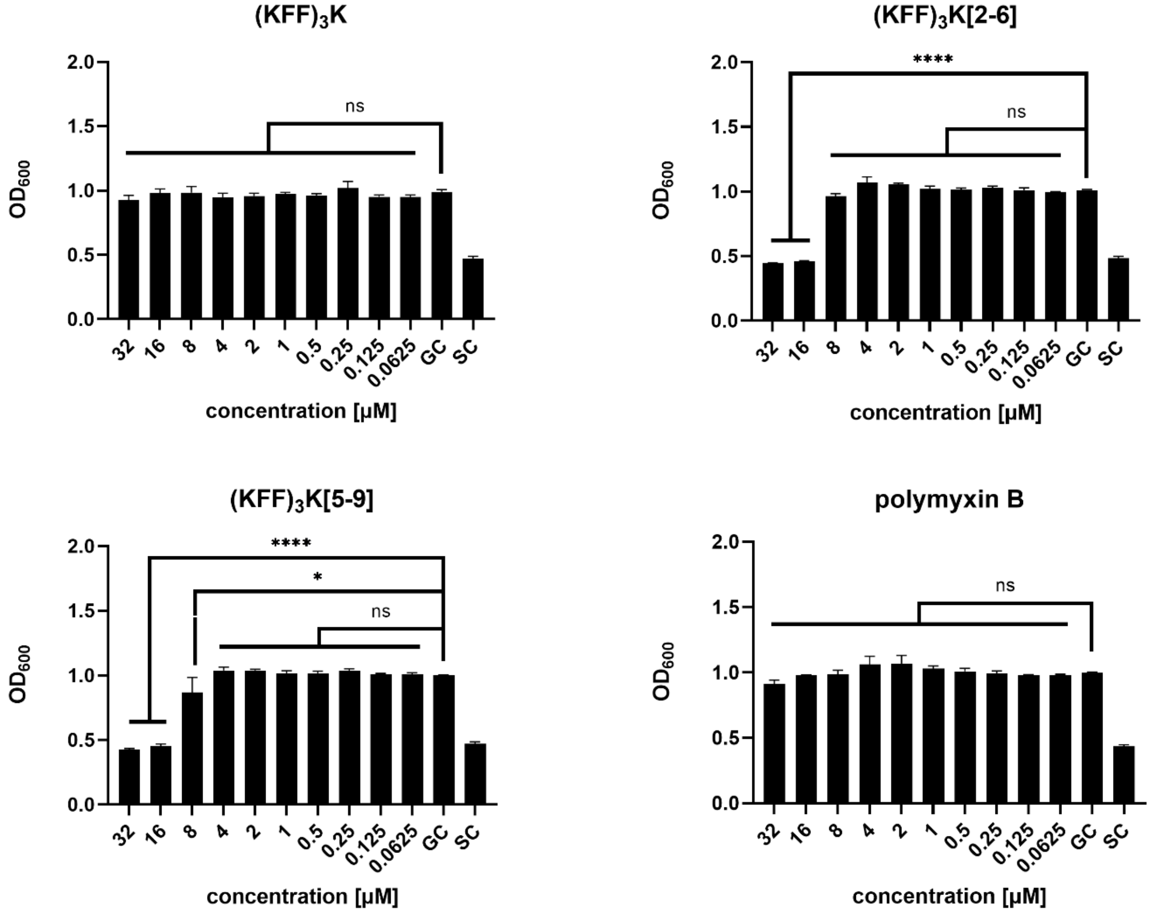

Supplementary Figure S15. The MIC results for the *S. aureus* ATCC 29213 strain. Non-stapled $(KFF)_3K$ together with stapled peptides and polymyxin B were tested in various concentrations. GC – growth control, SC – sterility control. For each sample experiment was repeated in two biological replicates of two technical replicates each. Error bars represent the standard error of the mean. Statistical significance between the samples and GC: **** P < 0.0001, * P <0.05, ns – not significant.

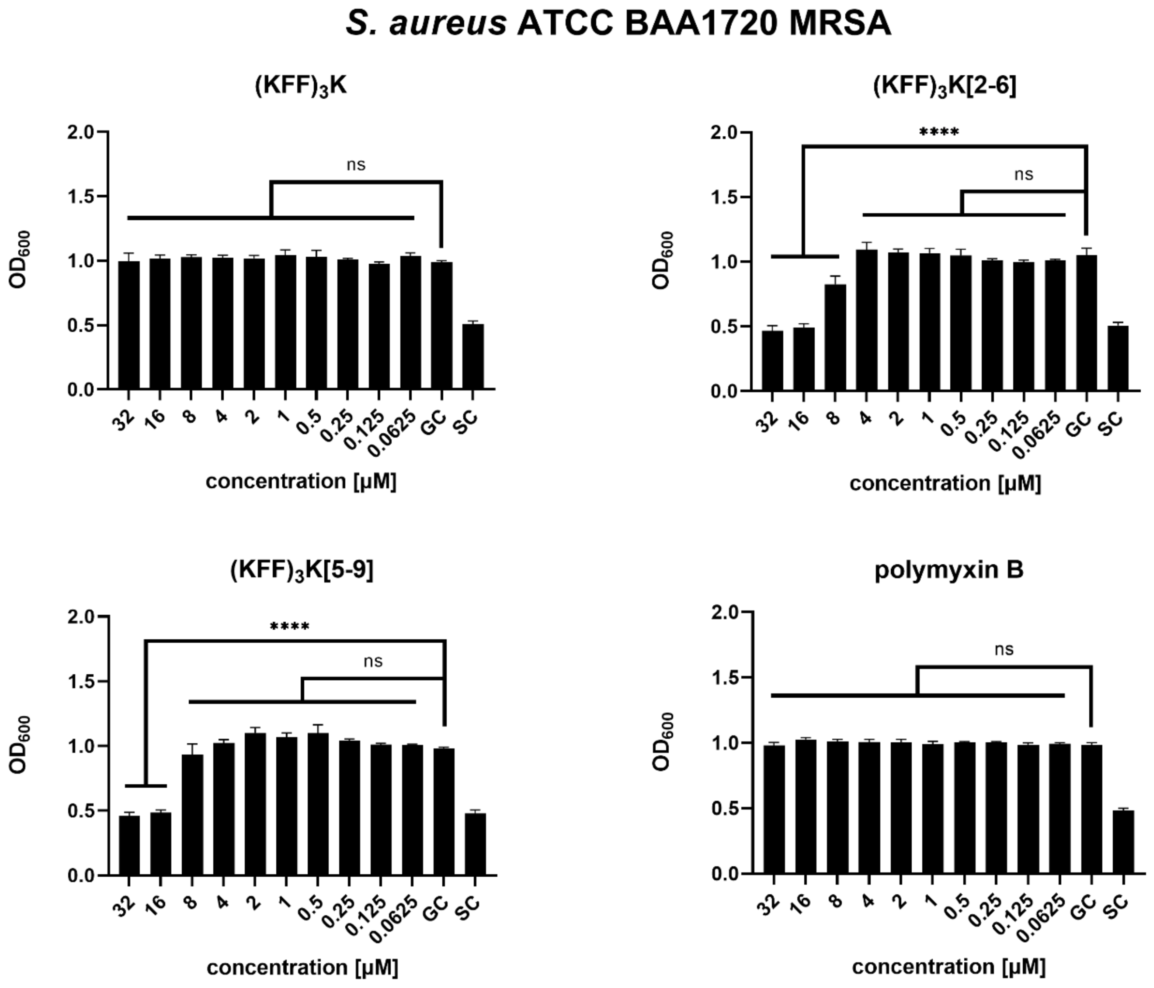

Supplementary Figure S16. The MIC results for the *S. aureus* ATCC BAA1720 MRSA strain. Non-stapled (KFF)$_3$K together with stapled peptides and polymyxin B were tested in various concentrations. GC – growth control, SC – sterility control. For each sample experiment was repeated in two biological replicates of two technical replicates each. Error bars represent the standard error of the mean. Statistical significance between the samples and GC: **** P < 0.0001, ns – not significant.
